# Histone chaperone-based stratification combined with two-sample Mendelian randomization identifies ADORA2B and SAPCD2 as prognostic biomarkers in esophageal cancer

**DOI:** 10.3389/fonc.2026.1764927

**Published:** 2026-04-13

**Authors:** Bihan Xia, Yuzhi Liu, Rui Zhao, Kai Deng

**Affiliations:** 1 Department of Gastroenterology and Hepatology, West China Hospital, Sichuan University, Chengdu, Sichuan, China; 2 Sichuan University-University of Oxford Huaxi Joint Centre for Gastrointestinal Cancer, Frontiers Science Center for Disease-Related Molecular Network, West China Hospital, Sichuan University, Chengdu, Sichuan, China; 3 Department of Endoscopy, Sichuan Clinical Research Center for Cancer, Sichuan Cancer Hospital & Institute, Sichuan Cancer Center, Affiliated Cancer Hospital of University of Electronic Science and Technology of China, Chengdu, China

**Keywords:** esophageal cancer, histone chaperone–related genes, Mendelian randomization, paclitaxel sensitivity, prognostic nomogram, regulatory T cells

## Abstract

**Background:**

Esophageal cancer (EC) lacks robust biomarkers to guide prognosis and therapy. Histone chaperone–related genes (HCRGs) shape chromatin states, but their roles in EC remain unclear. We integrated histone chaperone–based transcriptomic stratification with two-sample Mendelian randomization (MR) to identify genes with genetic evidence consistent with EC susceptibility and clinical relevance.

**Methods:**

RNA-seq and clinical data from TCGA-ESCA (184 tumors with available RNA-seq data and 13 normal samples) and an external cohort (GSE53624; 119 tumors, 119 normals) were analyzed. Prognosis-associated HCRGs informed unsupervised clustering. Differentially expressed genes common to cluster and tumor-versus-normal contrasts entered a two-sample MR framework using eQTL instruments and an EC GWAS (998 cases, 475,308 controls; European ancestry). Sensitivity analyses and the Steiger directionality test were then performed, followed by expression analysis to identify hub genes. We assessed survival associations, performed functional enrichment and immune deconvolution (CIBERSORT), modeled drug sensitivity (GDSC-based prediction), built a clinicogenomic nomogram, and validated expression by RT-qPCR in paired tissues.

**Results:**

HCRG-based clustering separated patients with distinct overall survival. MR implicated 26 genes in EC risk; among these, ADORA2B and SAPCD2 were consistently overexpressed in tumors (external validation and RT-qPCR) yet higher tumor expression predicted longer survival. Both genes were linked to cell-cycle and spliceosome programs; ADORA2B showed additional immuno-metabolic enrichment, whereas SAPCD2 mapped to metabolic/proteostasis pathways. High-expression groups exhibited reduced regulatory T-cell proportions and broader immune shifts. Predicted drug response suggested greater sensitivity to vinorelbine/etoposide but reduced sensitivity to paclitaxel in ADORA2B/SAPCD2-high tumors. A prognostic nomogram combining gene expression with stage and nodal status achieved 1–3-year AUCs of 0.67–0.75.

**Conclusions:**

Integrating HCRG-guided stratification with MR nominates ADORA2B and SAPCD2 as MR-supported biomarkers that are overexpressed in EC yet mark favorable prognosis, a pattern consistent with a less immunosuppressive microenvironment and distinct chemosensitivity. These genes warrant histology-stratified validation and mechanistic studies and may aid risk stratification and therapeutic decision-making.

## 1 Introduction

Esophageal cancer (EC) is the seventh most common malignancy worldwide, with over 470,000 new cases diagnosed each year (1). It ranks as the sixth leading cause of cancer-related mortality globally. EC primarily manifests in two histological subtypes: esophageal adenocarcinoma (EAC) and esophageal squamous cell carcinoma (ESCC), each with distinct etiologies and risk factors (2). Globally, ESCC accounts for nearly 90% of cases, with the highest burden observed in South America and across the so-called “esophageal cancer belt,” extending from East Africa through sub-Saharan regions to Central Asia. By contrast, EAC predominates in Europe and affluent areas of North America, where its incidence has risen approximately fourfold in the past forty years (3). The pathogenesis of EC involves a complex interplay of genetic, environmental, and lifestyle factors. Key risk factors include smoking, alcohol consumption, gastroesophageal reflux disease, and dietary habits. Despite advancements in diagnostic and therapeutic strategies, including surgical resection, chemotherapy, radiotherapy, and targeted therapies, the five-year survival rate for esophageal cancer remains low, ranging from 10% to 30% in most countries (4, 5). This persistently poor prognosis underscores the importance of identifying causal factors and understanding the molecular mechanisms underlying EC. Such insights are crucial for developing more effective prognostic markers and therapeutic targets, which could significantly improve patient outcomes.

Histone chaperones are essential for chromatin dynamics, ensuring correct nucleosome assembly/disassembly during DNA replication, repair, and transcription (6). By precisely guiding histone placement and preventing non-specific interactions, they maintain genomic stability and regulate gene expression (7). Consequently, dysfunction in histone chaperones contributes to cancer development and progression (8). For instance, APLF is overexpressed in triple-negative breast cancer (TNBC), promoting EMT to drive tumor invasion and metastasis, positioning it as a biomarker/therapeutic target (9). In breast cancer, metastatic stimuli suppress CAF-1, reducing H3.1/H3.2 deposition while increasing H3.3 incorporation—triggering chromatin remodeling and metastatic gene activation (10). In hepatocellular carcinoma (HCC), the FACT complex mediates oxidative stress responses via NRF2 feedback, promoting progression; its inhibitor Curaxin shows therapeutic promise (11). CRISPR screens reveal Asf1a enhances anti-PD-1 efficacy in KRAS-mutant lung adenocarcinoma (LUAD). Asf1a deficiency promotes macrophage differentiation and T-cell activation, suggesting its inhibition could improve immunotherapy outcomes (12). In clear cell renal cell carcinoma (ccRCC), SETD2 loss alters the epigenetic landscape, creating dependencies on ASF1A/ASF1B and SPT16 chaperone complexes to drive metastasis, highlighting them as therapeutic targets (13). However, the specific roles and mechanisms of histone chaperone-related genes (HCRGs) in EC remain insufficiently explored.

Mendelian Randomization (MR) is an analytical method utilizing genetic variants as instrumental variables to estimate the causal relationships between exposures and outcomes, mitigating confounding and reverse causation commonly observed in observational studies (14). This approach predominantly leverages genome-wide association studies (GWAS) and expression quantitative trait loci (eQTL) data to establish robust causal inferences (15). MR evaluates the causal effect by examining if genetic variants associated with an exposure also consistently correlate with the outcome (16). In a two-sample MR approach, genetic variants associated with an exposure in one dataset are tested for their effects on an outcome in a separate dataset, which enhances the robustness and reliability of causal inference by allowing for the examination of independent samples (17). Despite its advantages, the application of MR to decipher interactions between histone chaperones and EC remains sparse, underscoring the necessity for comprehensive research in this area.

The current study aimed to elucidate the potential causal relationships between HCRGs and EC using publicly available databases and a two-sample MR approach. Here, the exposures were the genetically predicted expression levels of HCRGs, derived from eQTL summary statistics, and the outcome was esophageal cancer risk from GWAS data. Specifically, we identified candidate genes with direct causal influence on EC and evaluated their prognostic significance. Furthermore, immune infiltration patterns, molecular regulatory networks, and drug sensitivity analyses associated with these biomarkers were explored to enhance understanding of EC pathogenesis and therapeutic potential. Overall, this study provides novel insights and valuable biomarkers that could facilitate improved diagnosis, prognosis, and targeted therapies for EC patients.

## 2 Methods

### 2.1 Data collection

The mRNA profile, clinic features (race, age at diagnosis, gender, stage, and Tumor-Node-Metastasis (TNM) staging), and overall survival (OS) of TCGA-Esophageal carcinoma (ESCA) were sourced from the Cancer Genome Atlas (TCGA, https://tcga-data.nci.nih.gov/tcga/). The TCGA-ESCA project includes 185 tumor cases and 13 solid tissue normal samples; however, RNA-seq expression data were available for 184 tumor samples, and therefore one tumor case without RNA-seq data was excluded from downstream transcriptomic analyses. Also, another mRNA profile of GSE53624 (GPL18109), derived from Asian (Chinese) individuals and comprising 119 EC patients and 119 normal controls was downloaded from the Gene Expression Omnibus (GEO) database (https://www.ncbi.nlm.nih.gov/geo/). Moreover, 30 HCRGs were gathered from the published literature (18). For the MR analysis, the GWAS data of EC (ebi-a-GCST90018841) was sourced from the Integrative Epidemiology Unit (IEU) Open GWAS database (https://gwas.mrcieu.ac.uk/). A total of 24,194,380 single nucleotide polymorphisms (SNPs) were obtained between EC (n=998) and normal controls (n=475,308), in which all the participants were of European ancestry. Furthermore, the eQTL data of common differently expressed genes (DEGs) was obtained also from the IEU Open GWAS database.

### 2.2 Unsupervised cluster analysis

In TCGA-ESCA, a total of 27 HCRGs were tested and listed in **Supplementary Table 1**. Based on the 27 HCRGs, the univariate Cox analysis was performed to screen the hub HCRGs related to prognosis using the survival (v. 0.4.9) R package (*p* < 0.2) (19). Subsequently, based on the hub HCRGs, the EC samples of TCGA-ESCA were clustered into distinct clusters by the ConsensusClusterPlus (v. 4.7.1.003) package (20). Using the survminer (v. 0.4.9) R package (https://CRAN.R-project.org/package=survminer), the Kaplan-Meier (K-M) curve was used to compare the OS difference between different subgroups via the Log-rank test (*p* < 0.05).

### 2.3 The differences in clinic features between the different subgroups

The expression levels of hub HCRGs in different clusters and clinic subgroups were shown by the heatmap using the ComplexHeatmap (v. 2.14.0) packages (21). Moreover, the proportions of the clinic features in different clusters were contrasted based on the chi-square test (*p* < 0.05).

### 2.4 Identification of DEGs

Using the DESeq2 (v. 1.42.0) package, the DEGs of different clusters were identified (|log_2_fold change (FC)| > 0.5, *p* < 0.05) (22). The volcano map and heat map of DEGs were drawn by the ggplot2 (v. 3.4.4) and ‘ComplexHeatmap (v. 2.14.0) packages, respectively (21, 23). Meanwhile, the DEGs between EC and normal controls in TCGA-ESCA were identified and displayed in the same ways as above (|log_2_FC| > 0.5, *p* < 0.05). Then, the common DEGs were obtained by overlapping the above two DEGs using the VennDiagram (v. 1.7.3) package (https://CRAN.R-project.org/package=VennDiagram).

### 2.5 Gene ontology and Kyoto encyclopedia of genes and genomes analyses

To further explore the potential function and signal pathways of common DEGs, the GO and KEGG analyses were performed by the clusterProfiler (v 4.10.0) package (adj.*p* < 0.05) (24). The top 10 GO items and the top 5 KEGG pathways were separately visualized via the ggplot2 (v. 3.4.4) (24).

### 2.6 Two-sample MR analysis

In two-sample MR, the screening of instrument variables (IVs) must meet the following conditions. On the one hand, IVs were not associated with confounding factors but strongly correlated with exposure factors. On the other hand, IVs must only affect outcome factors through exposure factors, but not directly. In this study, the two-sample MR was performed by the TwoSampleMR (v. 0.5.6) package (25), in which common DEGs were exposure factors (genes) and EC was an outcome factor. Importantly, IVs with strongly correlated exposure factors (genes) were screened (*p* < 10^-6^); IVs with linkage disequilibrium (LD) were filtered out (clump = TRUE, r^2^ = 0.001, kb = 10,000); weak instruments were excluded (F < 10), and exposures with fewer than three independent SNPs were not taken forward. Later, IVs that affected directly EC were eliminated based on the PhenoScanner database (26). The 5 algorithms (MR-Egger test (27), Weighted median (28), Inverse variance weighted (IVW) test (16), Simple mode (25), and Weighted mode (29)) were applied for two-sample MR analysis, and the IVW test was the main method in this study (*p* < 0.05). These complementary methods were selected to assess result robustness and detect potential violations of MR assumptions: MR-Egger regression tests for directional pleiotropy via its intercept test, while the weighted median can provide consistent estimates even if up to 50% of the weight comes from invalid instruments, whereas mode-based estimators are robust when the largest group of instruments with similar effects is valid. Consistency across multiple methods strengthens the reliability of inferred causal relationships. In particular, odds ratio (OR) value greater than 1 indicated a risk factor for EC, and vice versa was a protective factor. Both the GWAS and eQTL summary statistics were derived from predominantly European ancestry populations in the IEU OpenGWAS resource, and the datasets are independent with no sample overlap. The results of the two-sample MR analysis were displayed by the scatter plots, forest plots, and funnel plots. Across the 1,742-gene MR screen, statistical significance was assessed at p < 0.05 without multiple-testing correction, consistent with an exploratory analysis strategy. For each exposure gene, we computed SNP-level F-statistics as F and reported the gene-level mean F-statistic.

### 2.7 Sensitivity analysis and MR Steiger filtering

Sensitivity analysis was conducted using the TwoSampleMR (v. 0.5.6) and MRPRESSO (v. 1.0) packages to ensure the robustness of the MR results. Heterogeneity and horizontal pleiotropy were assessed using the mr_heterogeneity and mr_pleiotropy_test functions, respectively (p > 0.05). Horizontal pleiotropy was further evaluated using the mr_presso function (applicable when the number of SNPs > 3), as described by Verbanck et al. (30). The robustness of the causal estimates was additionally confirmed via leave-one-out (LOO) analysis using the mr_leaveoneout function, where the IVW estimate was recalculated iteratively after removing one SNP at a time. Finally, MR Steiger filtering was applied to confirm the directionality of causality, ensuring that the exposure factors (genes) influenced the outcome (esophageal cancer) rather than vice versa. Genes that passed all sensitivity and directionality tests were retained for downstream analyses.

### 2.8 Identification of candidate genes

In TCGA-ESCA, the expression levels of exposure factors (genes) were compared between EC and normal controls by the Wilcoxon test (*p* < 0.05). Subsequently, the consistency of the attributes of exposure factors (genes) and the expression trends was concerned. More specifically, as a risk factor, its expression level in EC samples was significantly higher than that of normal controls. On the contrary, as a protective factor, its expression level in EC samples was significantly lower than that of normal controls. Genes that met the above conditions were selected as candidate genes. Next, according to the optimal expression threshold of candidate genes, all EC samples in TCGA-ESCA were divided into high- and low-expression subgroups. K-M curves of each candidate gene were performed to compare the OS differences between high- and low-expression subgroups by the log-rank test (*p* < 0.05). In GSE53624, the expression levels of candidate genes with *p* < 0.05 were contrasted between EC and normal controls by the Wilcoxon test (*p* < 0.05). Then, candidate genes with significantly different expression levels and consistent with the trend of TCGA-ESCA were used as hub genes (*p* < 0.05). At last, the risk curves and survival states were displayed based on the high- and low-expression subgroups of hub genes.

### 2.9 Associations between different clinical subgroups and survival probability

According to age at diagnosis (less than 80, more than 80), gender (female, male), stage (stage i-ii, stage iii-iv), pathologic T (pT) stage (T1-T2, T3-T4), pN stage (N0-N3), and pM stage (M0, M1), the survival probabilities of different subgroups were compared by the log-rank test (*p* < 0.05). Afterwards, the clinic features with significant differences were selected as key features. Then, the rms (v. 6.5-0) package (https://CRAN.R-project.org/package=rms), the nomogram was constructed based on the hub features and genes to predict the 1-, 2-, and 3-year survival probability of EC patients. Finally, receiver operating characteristic (ROC) and calibration curves were used to evaluate the performance of the nomogram.

### 2.10 Gene set enrichment analysis

The *c2.cp.kegg.v2023.1.Hs.symbols.gmt* was downloaded from the Molecular Signatures Database v7.1 (MSigDB, https://www.gsea-msigdb.org/gsea/msigdb) database as a background gene set. Whereafter, spearman’s correlation coefficient between hub genes and other genes was calculated and ranked. To explore the biological functions of hub genes, the GSEA was performed by the clusterProfiler (v. 4.7.1) package (calculated nominal (NOM) p-value < 0.05, false discovery rate (FDR) q-value < 0.25) (24).

### 2.11 Tumor microenvironment

Based on the CIBERSORT, the 22 immune proportions of EC samples in TCGA-ESCA were calculated, and the samples with *p* < 0.05 were retained. The relative proportion of 22 immune cells in high- and low-expression subgroups of hub genes were shown as stacked bar charts. Meanwhile, the proportion differences were compared between high- and low-expression subgroups using the Wilcoxon test (*p* < 0.05). Additionally, spearman’s correlation analysis was conducted between hub genes and different immune cells (|cor| > 0.3, *p* < 0.05).

### 2.12 Molecular regulatory networks

After identifying the hub genes, the upstream molecular regulatory network was further explored. On the NetworkAnalyst database (https://www.networkanalyst.ca), the miRNAs related to hub genes were predicted, and the specific parameters were Specify organism: human, Gene-miRNA interaction database: miRTarBase v8.0. Subsequently, the top 5-degree miRNAs with the number of connections were used to predict the lncRNAs based on the miRNet (https://www.mirnet.ca/miRNet/home.xhtml) database. Next, the top 10-degree lncRNAs with the number of connections were selected. In the end, a lncRNA-miRNA-mRNA network was built based on the top 10-degree lncRNAs, top 5-degree miRNAs, and hub genes using the Cytoscape (v. 3.9.1) software (https://cytoscape.org/). Additionally, the transcription factors (TFs) related to hub genes were predicted from the JASPAR CORE database (http://jaspar.genereg.net/), and a TF-mRNA network was established using the Cytoscape (v. 3.9.1) software.

### 2.13 Drug sensitivity analysis

From the Genomics of Drug Sensitivity in Cancer (GDSC) database (https://www.cancerrxgene.org/), the biochemical half maximal inhibitory concentration (IC_50_) values of 138 drugs were downloaded. The pRRophetic package (v. 0.5) was used to estimate IC50 values for EC samples (31). The IC_50_ differences of drugs in high- and low-expression subgroups of hub genes were compared by the Wilcoxon test (adj.*p* < 0.05).

### 2.14 Clinical samples’ verification of hub genes

A total of 21 pairs of EC tissues and matched adjacent normal tissues were collected from Chinese patients undergoing surgery at West China Hospital of Sichuan University, with approval from the institutional ethics committee (No.179, 2021). Samples were snap-frozen and stored at -80 °C until analysis. Total RNA was extracted using TRIzol reagent (Invitrogen, Carlsbad, CA, USA) and reverse-transcribed into cDNA with the PrimeScript RT kit (Vazyme, Nanjing, China). Quantitative PCR was performed with SYBR Green Master Mix (Vazyme, Nanjing, China). Relative expression levels of signature genes were calculated by the 2^−ΔΔCt method, using GAPDH as an internal control. Differences between paired tumor and adjacent tissues were evaluated using paired t-tests. Primer sequences are provided in **Supplementary Table 2**, and the RT-qPCR validation results are presented in the Results section.

### 2.15 Statistical analysis

All statistical analyses were carried out by the R (v. 4.2.2) software. *P <* 0.05 was considered statistically significant in all conditions (two-tailed). This study follows the STROBE-MR reporting guidelines.

## 3 Results

### 3.1 A total of 1,742 common DEGs were identified

**Figure 1:**
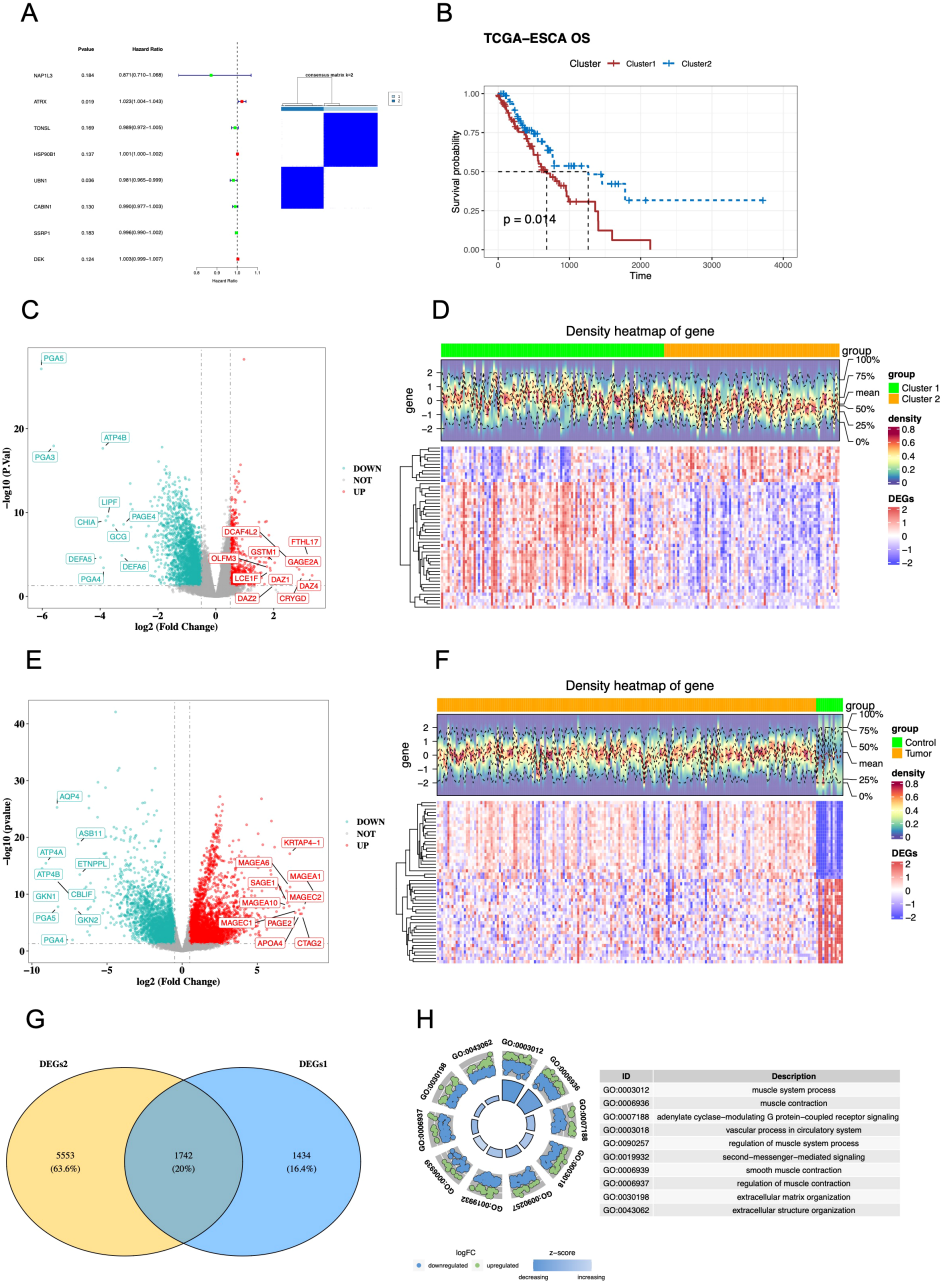
Histone chaperone-related molecular subtypes and common DEGs in EC. **(A)** Univariate Cox regression identified eight prognostic HCRGs (p < 0.2), which were used for consensus clustering of TCGA-ESCA tumors (n = 184); the consensus matrix supported k = 2. **(B)** Kaplan–Meier curves of OS between clusters (Cluster 1, n = 103; Cluster 2, n = 81; log-rank p = 0.014). **(C)** Volcano plot of DEGs between clusters (|log2FC| > 0.5; p < 0.05; DESeq2). **(D)** Heatmap of the top 50 cluster DEGs (row-wise z-scores). **(E)** Volcano plot of DEGs between tumors (n = 184) and normal tissues (n = 13) (|log2FC| > 0.5; p < 0.05). **(F)** Heatmap of the top 50 tumor–normal DEGs (row-wise z-scores). **(G)** Venn diagram intersecting cluster DEGs and tumor–normal DEGs, yielding 1,742 common DEGs. **(H)** GO enrichment of the common DEGs (top 10 terms; adj. p < 0.05).

Using the Cox analysis, there were 8 hub HCRGs related to prognosis of EC patients (*p* < 0.2) (**Supplementary Figure 1A**). Based on the hub HCRGs, the EC samples in TCGA-ESCA were divided into 2 clusters (k=2), namely cluster1 (n=103) and cluster2 (n=81) (**Figure 1A**). Importantly, the survival probability in cluster 1 was much worse than that of cluster 2 (**Figure 1B**). The expression levels of hub HCRGs in clusters and clinic subgroups were shown in **Supplementary Figure 1B**. The proportion of each clinical subgroup in the 2 clusters was calculated (**Supplementary Figure 1C**). Subsequently, there were 3,176 DEGs between cluster 1 and cluster 2, of which 655 up-regulated DEGs and 2,521 down-regulated DEGs (**Figures 1C, D**). Also, there were 7,295 DEGs between EC and normal controls which contained 3,954 up-regulated DEGs and 3,341 down-regulated DEGs (**Figures 1E, F**). By overlapping, a total of 1,742 common DEGs were identified (**Figure 1G**). Moreover, the GO results showed that common DEGs were associated with the muscle system process, muscle contraction, adenylate cyclase-modulating G protein-coupled receptor signaling pathway, etc. (**Figure 1H**; **Supplementary Table 3**). And the KEGG results indicated that common DEGs were linked with the neuroactive ligand-receptor interaction, dilated cardiomyopathy, cAMP signaling pathway, etc. (**Supplementary Figure 1D**; **Supplementary Table 4**).

### 3.2 Two-sample MR prioritized 26 susceptibility-associated genes for EC

**Figure 2:**
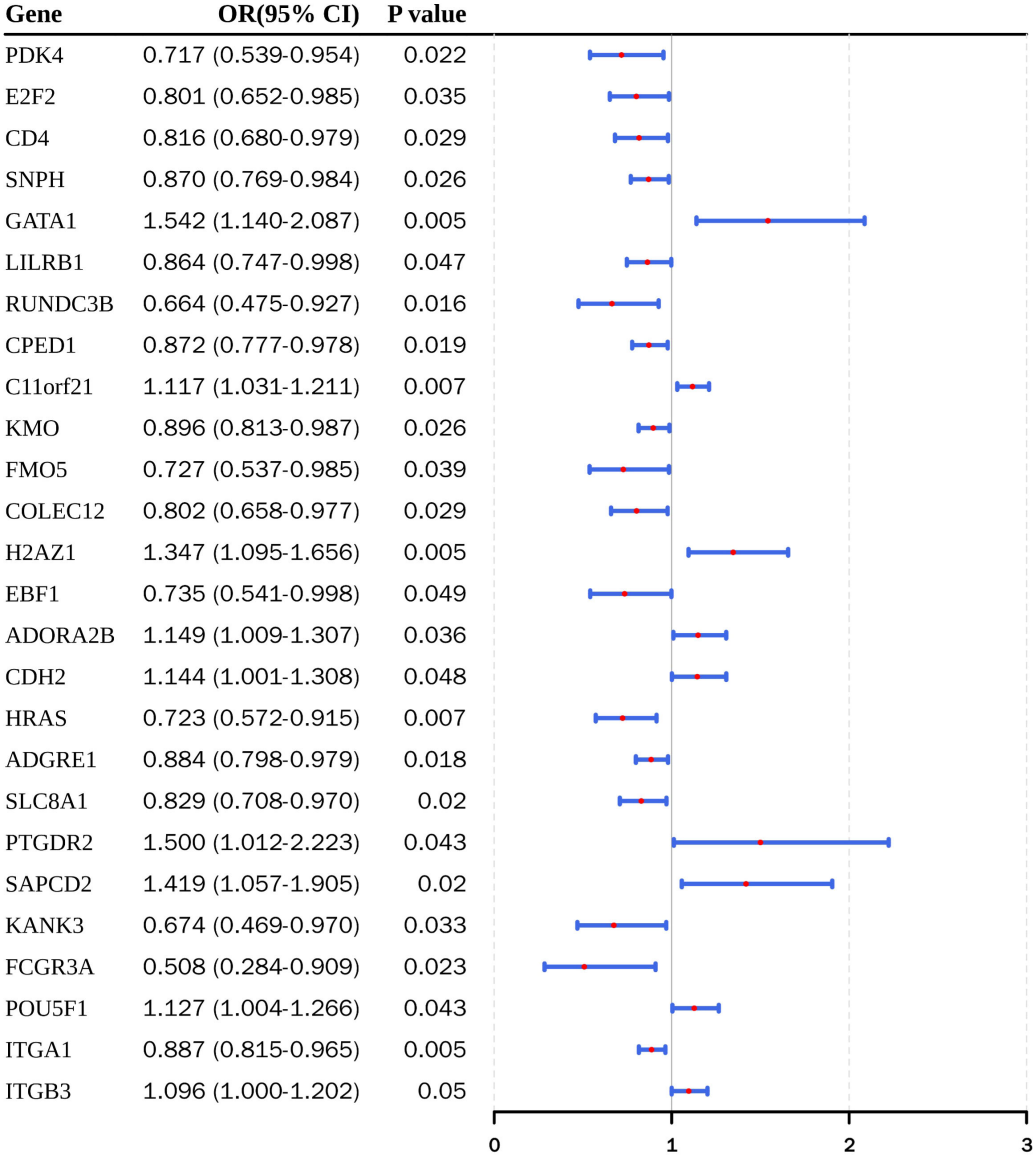
Two-sample MR of common DEGs implicates 26 genes in EC risk.

In the two-sample MR analysis, the eQTL data of 1,742 common DEGs were exposure factors (genes), and the EC was an outcome factor. After screening effective IVs, the two-sample MR analysis was performed to screen the exposure factors (genes) for EC occurrence. According to the IVW test, there were 26 exposure factors (genes) were determined, including 9 risk factors (*H2AZ1*, *ADORA2B*, *SAPCD2*, *ITGB3*, *GATA1*, *C11orf21*, *CDH2, PTGDR2*, and *POU5F1*) (OR > 1) and 17 protective factors (*FCGR3A*, *RUNDC3B*, *KANK3*, *PDK4*, *HRAS*, *FMO5*, *EBF1*, *E2F2*, *COLEC12*, *CD4*, *SLC8A1*, *LILRB1*, *SNPH*, *CPED1*, *ADGRE1*, *ITGA1*, and *KMO*) (OR < 1) (**Figure 2**; **Supplementary Table 5**).

Forest plot of IVW odds ratios (OR) with 95% CIs for 26 MR-implicated genes prioritized from 1,742 common DEGs. Red dots indicate IVW ORs and blue horizontal bars represent 95% CIs; the vertical reference line denotes OR = 1. OR > 1 indicates increased EC risk and OR < 1 indicates a protective association. P values are listed on the left. Full MR estimates across methods and sensitivity analyses are provided in **Supplementary Table 5**, **Supplementary Figures S2**–**S7**.

In scatter plots, the slopes of risk factors were positive in the IVW, and the slopes of protective factors were negative (**Supplementary Figures S2**, **S3**). In forest plots, the IVs of risk factors were on the right side of the line in the IVW test, and the IVs of protective factors were on the left side of the line (**Supplementary Figures S4**, **5**). Furthermore, the IVs of 26 exposure factors (genes) were distributed symmetrically along both sides of the IVW line, respectively (**Supplementary Figure 6**). No evidence of heterogeneity or directional pleiotropy was detected in the sensitivity tests (**Supplementary Tables S6**, **S7**). LOO analyses were performed to assess the influence of individual instruments, with detailed SNP-omission estimates provided (**Supplementary Table 12**, **Supplementary Figure 7**). We additionally report the final number of independent SNP instruments retained for each gene (**Supplementary Table 10**) and the gene-level mean F-statistics summarizing instrument strength (**Supplementary Table 11**). MR-Steiger directionality testing further supported the hypothesized causal direction for the 26 genes (**Table 1**).

**Table 1:** MR-Steiger directionality test results for 26 MR-implicated genes.

id.exposure	symbol	r2.exp	r2.out	correct	steiger_p
eqtl-a-ENSG00000004799	PDK4	0.022	2.78E-05	TRUE	2.29E-123
eqtl-a-ENSG00000007968	E2F2	0.025	3.72E-05	TRUE	5.33E-141
eqtl-a-ENSG00000010610	CD4	0.031	1.83E-05	TRUE	1.43E-190
eqtl-a-ENSG00000101298	SNPH	0.032	1.05E-05	TRUE	7.24E-191
eqtl-a-ENSG00000104972	LILRB1	0.061	1.11E-05	TRUE	4.10E-307
eqtl-a-ENSG00000106034	CPED1	0.064	1.99E-05	TRUE	0.00E+00
eqtl-a-ENSG00000131781	FMO5	0.038	1.34E-05	TRUE	7.33E-90
eqtl-a-ENSG00000158270	COLEC12	0.032	2.00E-05	TRUE	2.94E-200
eqtl-a-ENSG00000164032	H2AZ1	0.05	1.74E-05	TRUE	2.32E-123
eqtl-a-ENSG00000164330	EBF1	0.008	1.05E-05	TRUE	1.14E-49
eqtl-a-ENSG00000170425	ADORA2B	0.04	1.14E-05	TRUE	4.71E-207
eqtl-a-ENSG00000170558	CDH2	0.064	4.28E-05	TRUE	0.00E+00
eqtl-a-ENSG00000174775	HRAS	0.035	1.69E-05	TRUE	2.01E-160
eqtl-a-ENSG00000183023	SLC8A1	0.039	1.67E-05	TRUE	1.04E-240
eqtl-a-ENSG00000186193	SAPCD2	0.139	2.11E-05	TRUE	0.00E+00
eqtl-a-ENSG00000203747	FCGR3A	0.016	1.13E-05	TRUE	2.32E-40
eqtl-a-ENSG00000213949	ITGA1	0.051	2.22E-05	TRUE	0.00E+00
eqtl-a-ENSG00000259207	ITGB3	0.152	3.38E-05	TRUE	0.00E+00
eqtl-a-ENSG00000102145	GATA1	0.036	2.34E-05	TRUE	1.30E-71
eqtl-a-ENSG00000105784	RUNDC3B	0.018	1.24E-05	TRUE	2.31E-95
eqtl-a-ENSG00000110665	C11orf21	0.068	2.05E-05	TRUE	8.63E-294
eqtl-a-ENSG00000117009	KMO	0.041	1.50E-05	TRUE	2.69E-257
eqtl-a-ENSG00000174837	ADGRE1	0.049	2.31E-05	TRUE	0.00E+00
eqtl-a-ENSG00000183134	PTGDR2	0.01	1.21E-05	TRUE	1.42E-48
eqtl-a-ENSG00000186994	KANK3	0.013	2.17E-05	TRUE	9.70E-50
eqtl-a-ENSG00000204531	POU5F1	0.217	1.03E-05	TRUE	4.36E-287

### 3.3 The nomogram was built based on ADORA2B, SAPCD2, stage, and pN stage

**Figure 3:**
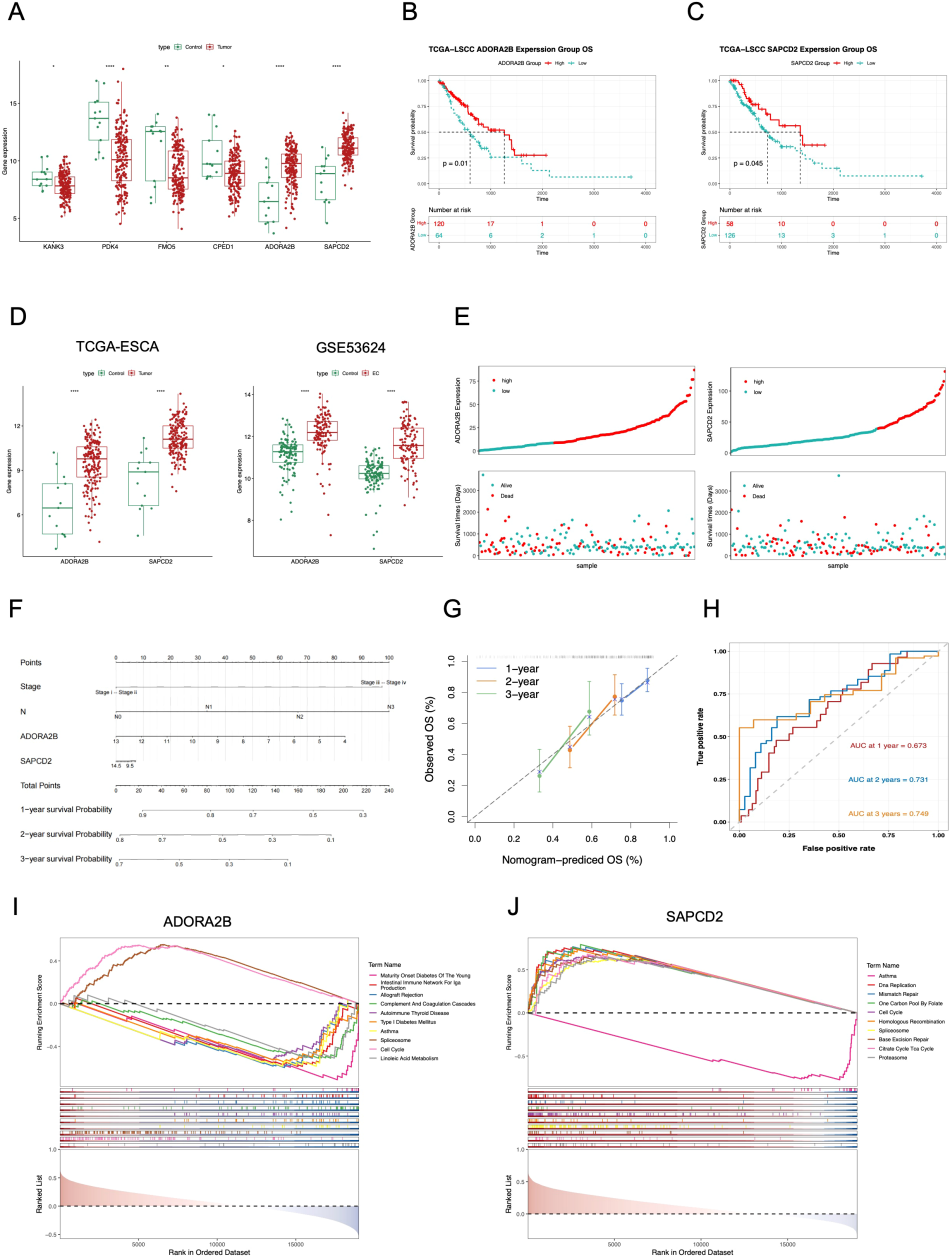
Validation and functional characterization of ADORA2B and SAPCD2 as prognostic biomarkers in ESCA. **(A)** Expression of six candidate genes in TCGA-ESCA (Wilcoxon, p < 0.05). **(B, C)** Kaplan–Meier OS curves for ADORA2B **(B)** and SAPCD2 **(C)** high/low groups defined by the optimal cut-off (survminer; log-rank p < 0.05). **(D)** External validation of ADORA2B/SAPCD2 overexpression in TCGA-ESCA (tumor vs normal) and GSE53624 (119 tumors/119 normals) (Wilcoxon, p < 0.05). **(E)** Expression-rank plots and survival status illustrating more deaths in the low-expression groups. **(F)** Prognostic nomogram integrating ADORA2B, SAPCD2, stage and nodal status; higher total points indicate higher risk. **(G)** Calibration for 1/2/3-year OS. **(H)** Time-dependent ROC of the nomogram (AUCs reported in the panel). **(I, J)** GSEA highlighting representative pathways enriched in the high-expression groups of ADORA2B **(I)** and SAPCD2 **(J)** (NOM p < 0.05; FDR q < 0.25).

The expression of 26 exposure factors (genes) in TCGA-ESCA was listed in **Supplementary Figure S8A**, and a total of 6 candidate genes matched the attributes of the exposure factors (**Figure 3A**). Based on the expression of 6 candidate genes, the EC samples in TCGA-ESCA were divided into high- and low-expression subgroups, respectively. Among them, for *ADORA2B* and *SAPCD2*, the survival probability of high-expression subgroups was better than that of low-expression subgroups (*p* < 0.05) (**Figures 3B, C**), and the others didn’t have significant differences (**Supplementary Figures 8B–E**). Hence, the expression levels of *ADORA2B* and *SAPCD2* in EC samples were higher than those of normal controls in both TCGA-ESCA and GSE53624, and they were determined as hub genes (**Figure 3D**). Notably, ADORA2B and SAPCD2 were each instrumented by 4 independent SNPs (**Supplementary Table 10**), with gene-level mean F-statistics of 427.63 and 89.28, respectively (**Supplementary Table 11**), indicating strong instruments. LOO analyses showed that the IVW direction remained unchanged for both genes (**Supplementary Figure 7**, **Supplementary Table 12**). IVW estimates with 95% CIs are provided in **Supplementary Table 5**. Then, risk curves and survival states indicated that the death cases of low-expression subgroups in *ADORA2B* and *SAPCD2* were much more (**Figure 3E**).

Eventually, the survival probability of pN stage subgroups had a significant difference, so was the stage subgroups (**Supplementary Figure 9**). Based on the *ADORA2B*, *SAPCD2*, stage, and pN stage, a nomogram was built (**Figure 3F**). Calibration curves indicated that the predicted OS was close to the actual OS (**Figure 3G**). The area under curve (AUC) values at 1–3 years of the ROC curve were 0.673-0.749, which suggested that the nomogram had a great predictive effectiveness (**Figure 3H**).

### 3.4 Functional analyses of *ADORA2B* and *SAPCD2*

Moreover, GSEA further elucidated pathways enriched by these hub genes. In the TCGA-ESCA dataset, ADORA2B was significantly enriched in 53 pathways, while SAPCD2 was significantly enriched in 35 pathways (**Supplementary Table 8**). Specifically, ADORA2B and SAPCD2 were both enriched in pathways such as “spliceosome”, “cell cycle”, and “asthma” (**Figures 3I, J**). Simultaneously, ADORA2B participated in “allograft rejection” and “linoleic acid metabolism” pathways (**Figure 3I**), whereas SAPCD2 enriched in “DNA replication”, “citrate cycle TCA cycle”, and “proteasome” pathways (**Figure 3J**). The enrichment results indicated that these hub genes might play important roles in tumorigenesis and progression, deepening our understanding of the biological significance of these genes and their potential implications in ESCA mechanisms.

### 3.5 Regulatory T cells were highly correlated with *ADORA2B* and *SAPCD2*

**Figure 4:**
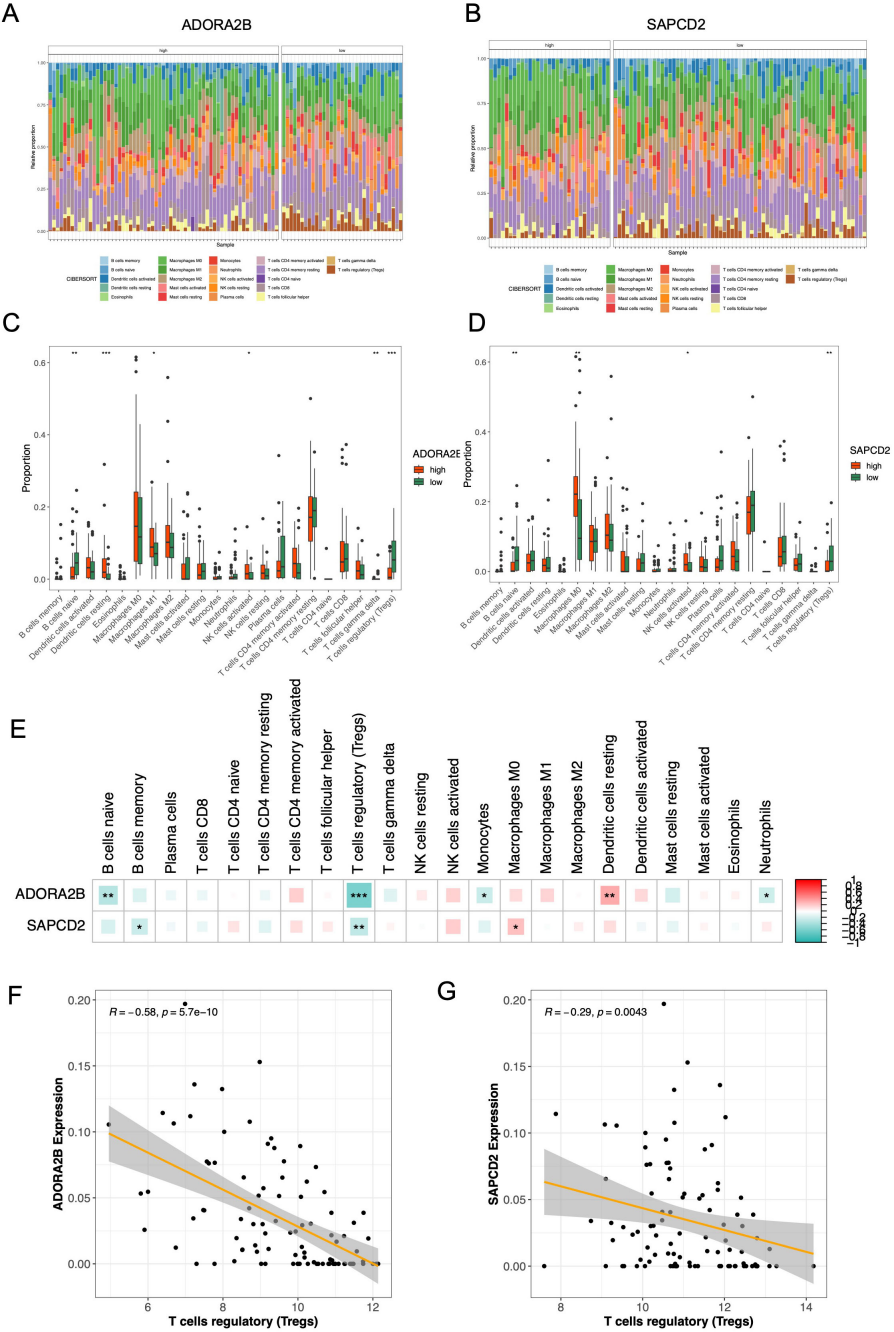
Immune microenvironment associations of ADORA2B and SAPCD2. **(A, B)** Stacked bar charts of the 22 CIBERSORT cell fractions in TCGA-ESCA tumors, split by ADORA2B **(A)** and SAPCD2 **(B)** expression groups (CIBERSORT, p < 0.05 retained). **(C, D)** Differences in immune-cell proportions between high vs. low expression groups (Wilcoxon, nominal p < 0.05). **(E)** Summary heatmap of significantly altered immune cells across both genes (asterisks indicate nominal significance). **(F, G)** Spearman correlations showing negative associations between gene expression and Tregs: ADORA2B r = −0.58, p = 5.7×10^-^¹^0^**(F)**; SAPCD2 r = −0.29, p = 0.0043 **(G)**.

After filtering outlier samples, a total of 96 EC samples in TCGA-ESCA were remained. And the relative proportion of 22 immune cells in high- and low-expression subgroups of *ADORA2B* and *SAPCD2* were shown in **Figures 4A, B**. Afterwards, 6 immune cells had significant differences between high- and low-expression subgroups of *ADORA2B*, and 4 immune cells had significant differences between high- and low-expression subgroups of *SAPCD2* (*p* < 0.05) (**Figures 4C, D**). Especially, naive B cells, activated natural killer (NK) cells, and Tregs were common different immune cells (**Figure 4E**). Interestingly, Tregs were highly negatively correlated with both *ADORA2B* (cor = -0.58, *p* < 0.05) and *SAPCD2* (cor = -0.29, *p* < 0.05), which may reflect the essential function of Tregs in EC tumor microenvironment (**Figures 4F, G**).

### 3.6 Construction of lncRNA–miRNA–mRNA (ceRNA) and TF–mRNA regulatory networks

Under the ceRNA hypothesis, lncRNAs may act as competing endogenous RNAs that sequester miRNAs and thereby de-repress their target mRNAs (32). This regulatory mode has been reported in esophageal cancer; for example, lncRNA HOTAIR can sponge miR-148a to relieve repression of Snail2 and promote EMT (33), and NORAD/miR-244-3p/MTDH axis can promote nuclear accumulation of β-catenin then lead to CDDP resistance of EC (34).

**Figure 5:**
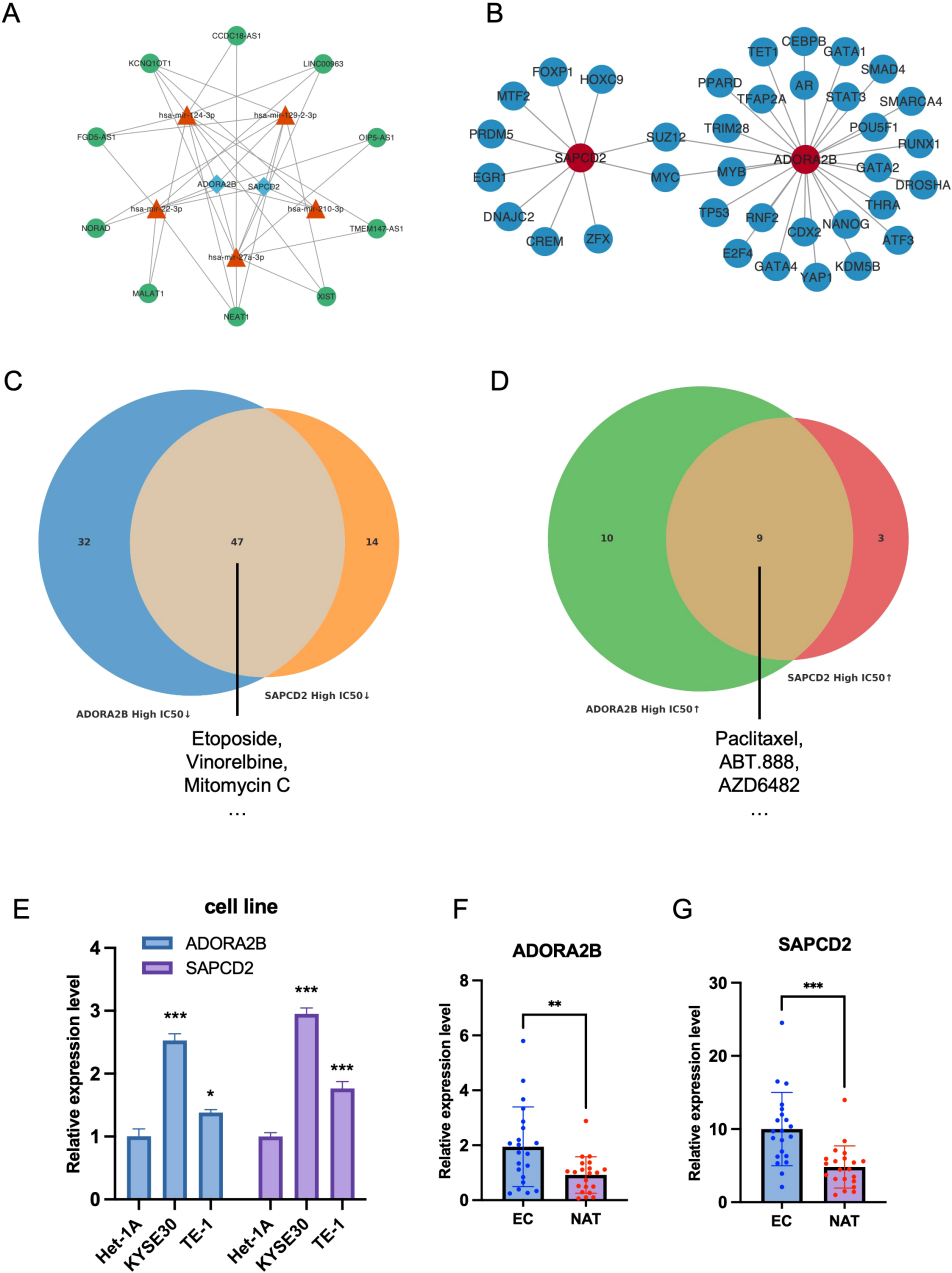
Regulatory networks, drug sensitivity, and experimental validation. **(A)** LncRNA–miRNA–mRNA (ceRNA) subnetwork from miRTarBase/miRNet. **(B)** TF–mRNA network for ADORA2B/SAPCD2 predicted from JASPAR. **(C, D)** Venn diagrams of drugs with lower **(C)** or higher **(D)** predicted IC50 in the high-expression groups of both genes (GDSC/pRRophetic; Wilcoxon, adj.p < 0.05); overlaps include etoposide, vinorelbine, mitomycin C **(C)** and paclitaxel **(D)**. **(E)** RT-qPCR in cell lines showing upregulated ADORA2B/SAPCD2 in KYSE30 and TE-1 versus Het-1A (t-test). **(F, G)** RT-qPCR in 21 paired primary EC tissues and matched NATs (paired t-test). *p < 0.05, **p < 0.01, ***p < 0.001.

Based on the *ADORA2B* and *SAPCD2*, a total of 50 miRNAs were predicted, and the top 5-degree miRNAs were selected for the subsequent analysis. Next, 160 candidate lncRNAs related to the top 5-degree miRNAs were predicted. Then, a lncRNA-miRNA-mRNA network was constructed using the top 10-degree lncRNAs, the top 5-degree miRNAs, and the 2 hub genes (**Figure 5A**). Notably, the selected high-degree miRNAs (hsa-miR-124-3p, hsa-miR-129-2-3p, hsa-miR-22-3p, hsa-miR-210-3p and hsa-miR-27a-3p) were simultaneously linked to both ADORA2B and SAPCD2, suggesting a shared upstream post-transcriptional regulatory module for the two prognostic biomarkers in EC. These interactions provide testable hypotheses and prioritize candidate miRNA/lncRNA regulators for future functional validation. Also, a total of 35 TFs related to hub genes were predicted on the JASPAR CORE database. A TF-mRNA network was built based on the these TFs and two hub genes. Notably, both SUZ12 and MYC were predicted to interacted with the hub genes, indicating potential common transcriptional regulation of these two biomarkers (**Figure 5B**). Moreover, the IC_50_ values of 98 and 73 drugs had significant differences between high- and low-expression subgroups of *ADORA2B* and *SAPCD2*, respectively (adj. *p* < 0.05) (**Supplementary Figures 10**, **11**). Notably, 47 drugs showed reduced IC50 values across both *ADORA2B*- and *SAPCD2*-high subgroups, among them several key agents in esophageal cancer therapy, including Etoposide, Vinorelbine, and Mitomycin C (**Figure 5C**; **Supplementary Table 9**). Conversely, 9 drugs demonstrated increased IC50 values, with Paclitaxel—a mainstay in esophageal cancer treatment—among them (**Figure 5D**; **Supplementary Table 9**).

### 3.7 Validation of *ADORA2B* and *SAPCD2* expression in cell lines and clinical specimens​​

**Figure 6:**
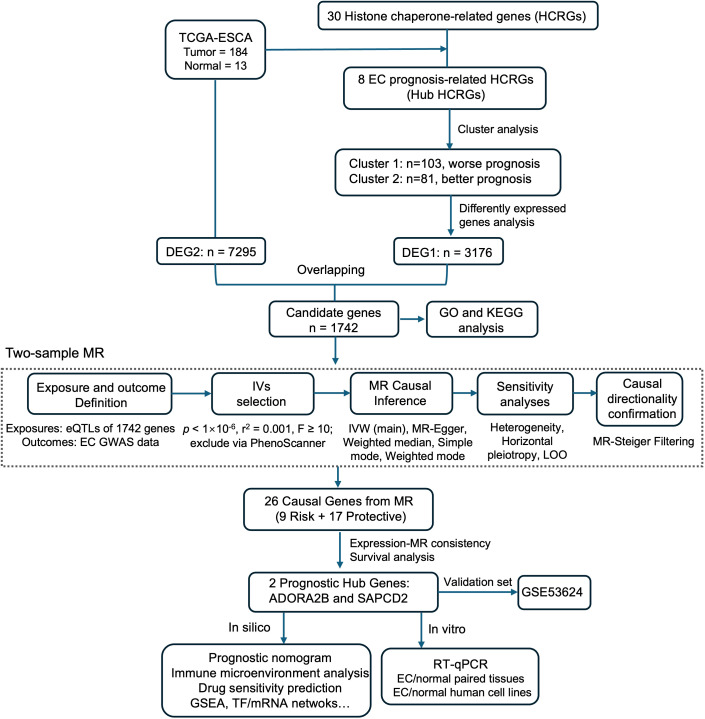
Schematic workflow of the study. From TCGA-ESCA (184 tumors/13 normals), 30 HCRGs were assessed; univariate Cox identified eight prognostic HCRGs, supporting k = 2 clustering with distinct OS. Intersecting DEGs from the cluster and tumor–normal contrasts yielded 1,742 genes; two-sample MR prioritized 26 EC risk–related genes. Consistency of expression direction and survival narrowed these to ADORA2B and SAPCD2, followed by downstream analyses and validation. Downstream modules include a well-calibrated clinicogenomic nomogram, immune profiling (higher ADORA2B/SAPCD2 linked to lower Tregs), and drug-response prediction (higher ADORA2B/SAPCD2 predicted reduced sensitivity to paclitaxel), plus pathway enrichment and regulatory networks, with external validation in GSE53624 and RT-qPCR in EC cell lines and 21 paired clinical tissues.

To further validate the bioinformatic findings of ADORA2B and SAPCD2 dysregulation in EC, RT-qPCR was performed in both cell lines and clinical samples. Expression of ADORA2B and SAPCD2 was measured in the normal human esophageal epithelial cell line Het-1A and in two esophageal cancer cell lines (KYSE30 and TE-1). Both genes were significantly upregulated in EC cell lines relative to Het-1A (p < 0.05 for each), with KYSE30 showing the highest expression among the tumor lines (**Figure 5E**). Consistent with these *in vitro* results and the preceding bioinformatic analyses, RT-qPCR of 21 paired primary EC tissues and matched adjacent non-tumorous tissues (NATs) confirmed marked overexpression of both genes in tumor tissues. Specifically, ADORA2B expression was significantly elevated in tumors compared with paired NATs (paired t-test, p < 0.05), while SAPCD2 was even more strongly overexpressed (paired t-test, p < 0.001) (**Figures 5F, G**). These results provide independent validation, in both cellular models and patient-derived tissues, that ADORA2B and SAPCD2 are aberrantly upregulated in EC, consistent with their identification as key factors in EC pathogenesis. A schematic summary of the analytical workflow, including prognostic HCRG screening, MR-based causal gene identification, and subsequent in silico and experimental validation, is shown in **Figure 6**.

## 4 Discussion

In this study, we combined transcriptome-based patient stratification and two-sample MR to identify and validate prognostic hub genes in esophageal cancer. By clustering patients on HCRG expression patterns and then applying MR analysis on differentially expressed genes, we uncovered ADORA2B and SAPCD2 as hub genes with genetic evidence consistent with potential causal involvement and prognostic relevance in EC. By integrating transcriptomic stratification with two-sample MR, our framework strengthens causal interpretation compared with expression–outcome associations alone. The identification of these two hub genes, neither of which is a canonical histone chaperones themselves, underscores how chromatin regulatory contexts can pinpoint downstream effectors of disease. Both ADORA2B and SAPCD2 were upregulated in tumor tissues, and their high expression was correlated with better patient survival, highlighting them as promising biomarkers for risk stratification and potential therapy targets.

ADORA2B (Adenosine A2B Receptor), a G-protein-coupled receptor, mediates adenosine-related signaling in hypoxic or inflammatory tumor microenvironments, thereby modulating cellular responses such as vasodilation, inflammation, and immune regulation (35, 36). ADORA2B has been reported to participate in pro-tumorigenic processes, in part by shaping the immune milieu. Mechanistically, adenosine-ADORA2B signaling can inhibit CD8+ T cell cytotoxicity, promote Tregs expansion, and polarize macrophages toward the M2 phenotype, collectively fostering immune evasion (37–39). Additionally, ADORA2B signaling is widely implicated in adenosine-mediated immunoregulation and can be pro-tumorigenic in many contexts (40, 41); however, prognostic associations of ADORA2B expression can be context-dependent and may reflect tumor ecosystem states, including immune infiltration and treatment responsiveness (42, 43). In esophageal cancer, several studies suggest that elevated ADORA2B may be associated with aggressive phenotypes and reduced immunotherapy efficacy, and experimental evidence indicates that ADORA2B can promote EC cell proliferation, migration, and invasion (44, 45). Notably, in our cohorts, although ADORA2B was upregulated in tumor tissues and validated by RT-qPCR, higher ADORA2B expression was associated with improved survival and reduced Tregs abundance, suggesting that ADORA2B may capture a clinically relevant, ecosystem-linked tumor state in established EC.

SAPCD2 (suppressor APC domain-containing 2), a gene involved in regulating cell proliferation and the cell cycle, has been reported to facilitate tumor progression and metastasis across multiple cancer types (46, 47). Previous literature documented SAPCD2’s involvement in modulating key oncogenic pathways such as Hippo/YAP, Wnt/β-catenin, and PI3K/AKT, essential for tumor cell proliferation, invasion, and metastasis across multiple malignancies (48–50). To date, the role of SAPCD2 in EC remains unreported. Remarkably, our study firstly identified SAPCD2 as a significantly upregulated gene in EC tissues. Collectively, ADORA2B and SAPCD2 function as critical hub genes potentially linked through common molecular mechanisms and signaling pathways, including PI3K/AKT and MAPK signaling, all well-documented in various cancers to facilitate tumor cell proliferation, survival, invasion, angiogenesis, and immune modulation (49, 51). Together with the shared upstream regulators identified in our network analyses, these results support a potentially coordinated regulatory module for the two biomarkers, which warrants future functional validation.

Our study also highlighted the shared and distinct pathways associated with ADORA2B and SAPCD2. The common enrichment of both genes in “cell cycle” and “spliceosome” pathways points to a convergence on fundamental processes of cell proliferation and RNA processing. Dysregulation of the spliceosome can profoundly affect gene expression programs in cancer, and it is intriguing that our hub genes correlate with this pathway, suggesting these tumors have altered mRNA splicing environments. Moreover, ADORA2B was uniquely enriched in pathways like allograft rejection and linoleic acid metabolism, whereas SAPCD2 was linked to TCA cycle and proteasome pathways. These differences suggest that both genes may be linked to a pro-proliferative state, while ADORA2B might interface more with immune-modulatory metabolic pathways (e.g. arachidonic/linoleic acid metabolites can influence inflammation), and SAPCD2 might more strongly influence core metabolic and proteostasis functions. This mechanistic divergence could underlie their combined yet non-identical effects on the tumor ecosystem.

Importantly, both genes were strongly associated with immune contexture changes, reinforcing that cell-intrinsic and extrinsic (immune) factors are intertwined in determining patient prognosis. In particular, the negative correlation between both ADORA2B/SAPCD2 and Tregs abundance is notable. Tregs, known to suppress anti-tumor immunity via secretion of cytokines (e.g., TGF-β, IL-10) and direct inhibition of effector T-cells, correlate with poor prognosis in EC (52, 53). Our data imply that high expression of these genes might discourage Tregs infiltration or maintenance, which could be beneficial for anti-tumor immune responses. Supporting this, recent research in ovarian cancer also found that low ADORA2B expression was linked to more aggressive disease and immunosuppression, consistent with our observation that ADORA2B (and by extension SAPCD2) high expressors had a less immunosuppressive TME (54). These findings raise the possibility that ADORA2B/SAPCD2 expression may track with an immune-active tumor state in EC. Whether this translates into differences in immunotherapy response requires validation in treatment-annotated cohorts and functional studies.

From a clinical translation standpoint, our construction of a prognostic nomogram integrating ADORA2B, SAPCD2, and key clinicopathological factors (stage and nodal status) could be valuable. The nomogram achieved appreciable accuracy (1–3 year AUCs of 0.67–0.75) in predicting survival, which is notable given the typically poor outcomes in EC. Such a tool could help clinicians in tailoring surveillance and treatment intensity – for example, identifying ostensibly high-risk patients who, by virtue of high ADORA2B/SAPCD2 expression, might actually have a better response to standard therapies and thus avoid overtreatment. Conversely, patients with low expression of these genes (and hence worse prognosis) might be triaged for more aggressive or novel treatments.

Drug sensitivity analyses further underscored the clinical relevance of these hub genes, suggesting that ADORA2B/SAPCD2-high tumors exhibited increased sensitivity to chemotherapeutic agents such as vinorelbine and etoposide, while showing resistance to paclitaxel. Vinorelbine, a microtubule-destabilizing agent that disrupts mitotic spindle formation, has demonstrated long-term clinical benefit when combined with cisplatin and radiotherapy as part of a neoadjuvant chemoradiotherapy (NCRT) regimen (55, 56). In a phase III randomized clinical trial, vinorelbine–cisplatin-based NCRT followed by surgery significantly improved both OS and disease-free survival (DFS) compared to surgery alone in patients with locally advanced ESCC (56). Etoposide, a topoisomerase II inhibitor that induces DNA strand breaks and apoptosis, can be used in small cell neuroendocrine carcinoma of the esophagus (SCNEC-E), a rare histological subtype (57, 58). Conversely, elevated ADORA2B/SAPCD2 expression was associated with reduced sensitivity to paclitaxel, a mitotic inhibitor that stabilizes microtubules and arrests cells in G2/M phase. It is a cornerstone of both neoadjuvant (e.g., paclitaxel–carboplatin regimens) and palliative chemotherapy for advanced ESCC, as endorsed by major guidelines such as National Comprehensive Cancer Network (NCCN) and European Society for Medical Oncology (ESMO) (59, 60). It might be that ADORA2B/SAPCD2-high tumors engage survival pathways that particularly counter microtubule stabilization by paclitaxel. If validated, this could guide a personalized approach: patients with high ADORA2B/SAPCD2 tumors could benefit from alternate regimens (substituting paclitaxel with vinorelbine, for instance). Additionally, ADORA2B is a druggable target – several antagonists and antibodies are in development or trials aiming to alleviate adenosine-mediated immunosuppression (61, 62). If our findings hold, one might envision a combined strategy where ADORA2B blockade is used to boost immune response while leveraging the inherent chemosensitivity of ADORA2B-high tumors to maximize tumor kill. As for SAPCD2, while not yet a direct drug target, its role in cell cycle regulation suggests that CDK inhibitors or mitotic checkpoint modulators could be explored in SAPCD2-driven tumors. Moreover, the upstream regulators we identified (e.g. miR-210-3p targeting ADORA2B, LINC01355 and others) might themselves serve as therapeutic entry points, such as using miRNA mimics or lncRNA inhibitors to modulate SAPCD2/ADORA2B expression.

A seemingly discordant pattern is observed in our results: MR suggests that genetically proxied higher expression of ADORA2B and SAPCD2 increases EC susceptibility, whereas in tumor cohorts both genes are upregulated and higher tumor expression is associated with better survival. This is not unexpected because these analyses address different endpoints at different disease stages. Specifically, two-sample MR leverages germline eQTL instruments to estimate the effect of lifelong, genetically proxied expression on EC liability in the general population, which is most relevant to tumor initiation. In contrast, tumor expression–survival associations are evaluated among diagnosed patients and thus reflect post-diagnosis outcomes shaped by tumor heterogeneity, microenvironmental states (including immune/stromal admixture), and treatment exposure. Accordingly, a susceptibility-increasing genetic effect can coexist with a favorable prognostic association if higher tumor expression marks an immune-active and/or therapy-responsive tumor state after cancer has developed. Consistent with this possibility, higher ADORA2B/SAPCD2 expression in our cohorts co-occurred with a distinct immune contexture (e.g., lower inferred Treg fractions) and differential predicted chemosensitivity (e.g., lower predicted IC50 for vinorelbine/etoposide and higher predicted IC50 for paclitaxel). Similar dissociations have been reported in other settings. For example, BRCA1/2 germline mutations markedly increase cancer risk yet have been linked to improved overall survival among ovarian cancer patients (63). A related “tumor-high yet better outcome” pattern has also been described in gastrointestinal cancers for therapy-sensitivity markers. Higher SLFN11 expression has been associated with improved survival, with stronger separation among patients receiving platinum-based chemotherapy in gastric cancer (64); and in pancreatic cancer, higher hENT1/SLC29A1 expression has been associated with improved benefit from gemcitabine-based therapy (65). Finally, we acknowledge that prognostic analyses conditioned on case status may also be influenced by index-event (collider) selection effects, which can further decouple incidence-oriented genetic associations from post-diagnosis outcomes (66).

Our study has several strengths, including Mendelian randomization–based causal inference and multi-dimensional validation (in silico, *in vitro*, and clinical tissues). Nevertheless, several limitations should be acknowledged. First, methodological limitations include the use of MR instruments derived predominantly from European-ancestry populations; although we included an independent Asian transcriptomic cohort (GSE53624) and a Chinese RT-qPCR validation set for expression-level confirmation, ancestry-matched MR replication in non-European datasets remains warranted. In addition, TCGA-ESCA includes mixed histologies, and histology-stratified validation will be useful. Moreover, clustering was performed using prognosis-selected HCRGs from the same cohort, which may introduce overfitting bias and should be validated using a prespecified HCRG set and external cohorts. Second, clinical-translational limitations apply to the downstream analyses. Immune deconvolution and drug-sensitivity prediction are based on computational inference and pharmacogenomic resources and therefore remain correlative and hypothesis-generating. Likewise, the nomogram and drug-response hypotheses were developed from retrospective data rather than regimen-matched clinical response datasets, and thus should be validated in independent cohorts with standardized treatment and outcome annotation prior to any potential clinical implementation. Third, mechanistic limitations remain. MR supports causal inference at the population level but does not establish tumor-level mechanisms; beyond expression validation by RT-qPCR, functional perturbation experiments in EC models will be needed to clarify the cell-intrinsic roles of ADORA2B and SAPCD2 and their potential links to tumor microenvironmental programs (e.g., immune-cell recruitment).

In conclusion, using an MR-guided integrative strategy, we prioritized ADORA2B and SAPCD2 as genetically supported hub genes that may contribute to EC susceptibility and stratify prognosis after diagnosis. Despite tumor upregulation, higher expression was linked to better survival, potentially aligning with a less immunosuppressive immune contexture and differences in predicted chemotherapy sensitivity. Together, our results highlight ADORA2B and SAPCD2 as candidate prognostic biomarkers and provide testable therapeutic hypotheses. Next steps should focus on validating the nomogram and drug-sensitivity signals in independent, treatment-annotated cohorts (preferably with regimen-specific endpoints) and on mechanistic experiments to disentangle tumor-intrinsic functions from microenvironment-associated signals. In particular, given the negative association with inferred Treg fractions, it will be informative to test whether these genes are linked to pathways regulating Treg recruitment or function, ideally using single-cell or spatial profiling followed by targeted perturbation.

## Data Availability

The datasets presented in this study can be found in online repositories. The names of the repository/repositories and accession number(s) can be found in the article/**Supplementary Material**.

## Supplementary material

Supplementary Table 1 Univariate Cox regression results of 27 histone chaperone–related genes in TCGA-ESCA.

Supplementary Table 2 Primer sequences used for RT-qPCR validation.

Supplementary Table 3 Gene Ontology enrichment results of common DEGs.

Supplementary Table 4 KEGG pathway enrichment results of common DEGs.

Supplementary Table 5 Mendelian randomization estimates for 26 MR-implicated genes associated with EC risk.

Supplementary Table 6 Heterogeneity test results of MR analyses.

Supplementary Table 7 Horizontal pleiotropy test results of MR analyses.

Supplementary Table 8 Gene set enrichment analysis of ADORA2B and SAPCD2.

Supplementary Table 9 Predicted drug sensitivity differences by ADORA2B and SAPCD2 expression.

Supplementary Table 10 SNP-level instrument information for the 26 MR-implicated genes.

Supplementary Table 11 Gene-level mean F-statistics for the 26 MR-implicated genes.

Supplementary Table 12 Leave-one-out sensitivity results for the 26 MR-implicated genes.

Supplementary Figure 1 Prognostic HCRGs, HCRG-based clustering, clinicopathologic distributions, and KEGG enrichment. (A) Univariate Cox regression identifies eight prognosis-related hub HCRGs in TCGA-ESCA (p < 0.20). (B) Heatmap of hub HCRG expression with sample clustering and clinical annotations. (C) Distribution of clinicopathologic features across the two clusters (χ²/Fisher’s exact tests). (D) KEGG enrichment of the 1,742 common DEGs (see **Supplementary Table 4** for full results).

Supplementary Figure 2 Scatter plots of two-sample MR for the 9 MR-implicated risk genes. Each dot represents one SNP instrument. The x-axis shows the SNP effect on gene expression (βX, eQTL), and the y-axis shows the SNP effect on EC risk (βY, GWAS; log-odds scale). Colored lines indicate causal estimates from different MR methods (IVW, MR-Egger, weighted median, simple mode, and weighted mode).

Supplementary Figure 3 Scatter plots of two-sample MR for the 17 MR-implicated protective genes. Each dot represents one SNP instrument. The x-axis shows the SNP effect on gene expression (βX, eQTL), and the y-axis shows the SNP effect on EC risk (βY, GWAS; log-odds scale). Colored lines indicate causal estimates from different MR methods (IVW, MR-Egger, weighted median, simple mode, and weighted mode).

Supplementary Figure 4 Single-SNP and pooled MR estimates for the 9 MR-implicated risk genes. For each gene, red points with horizontal lines indicate single-SNP Wald ratio estimates with 95% CIs. Blue points/lines at the bottom show pooled estimates from All–IVW (primary) and All–MR-Egger (sensitivity). The vertical dashed line denotes the null (β = 0, equivalent to OR = 1).

Supplementary Figure 5 Single-SNP and pooled MR estimates for the 17 MR-implicated protective genes. For each gene, red points with horizontal lines indicate single-SNP Wald ratio estimates with 95% CIs. Blue points/lines at the bottom show pooled estimates from All–IVW (primary) and All–MR-Egger (sensitivity). The vertical dashed line denotes the null (β = 0, equivalent to OR = 1).

Supplementary Figure 6 Funnel plots for MR analyses of the 26 MR-implicated genes.For each gene, the x-axis shows the SNP-specific causal estimate (Wald ratio), and the y-axis shows its precision (1/SE). Funnel plot symmetry provides a visual check for potential directional pleiotropy or outlier-driven asymmetry; formal pleiotropy testing results (MR-Egger intercept) are provided in **Supplementary Table 7**.

Supplementary Figure 7 Leave-one-out (LOO) sensitivity analyses for MR robustness. For each gene, LOO analysis evaluates whether the IVW estimate is disproportionately driven by a single SNP. The blue point/line denotes the IVW estimate using all SNP instruments (with 95% CI) as the baseline. Each red point/line shows the IVW estimate recalculated after removing one SNP in turn. The vertical dashed line indicates the null (β = 0; OR = 1).

Supplementary Figure 8 Expression patterns and survival analyses of additional MR-implicated genes. (A) Expression levels of the 26 MR-implicated genes in TCGA-ESCA tumor versus normal tissues. Group differences were evaluated using a two-sided Wilcoxon rank-sum test; significance is indicated by asterisks. (B–E) Kaplan–Meier overall survival (OS) analyses for representative MR-implicated genes that did not reach significance in the main candidate-gene survival results. Patients were stratified into high- and low-expression groups (as defined in the Methods); log-rank p values are shown.

Supplementary Figure 9 Survival analyses in clinicopathologic subgroups of TCGA-ESCA.Kaplan–Meier OS curves stratified by clinicopathologic subgroups (e.g., stage, T/N category, or other variables as specified) are shown. Log-rank p values are provided to assess survival differences within each subgroup.

Supplementary Figure 10 Predicted drug sensitivity stratified by ADORA2B expression. Predicted IC50 values were inferred from TCGA-ESCA transcriptomes. Patients were divided into high and low expression groups for ADORA2B. Each panel shows one drug, with violin/box plots comparing predicted IC50 between groups (lower IC50 indicates higher sensitivity); group differences were tested using a two-sided Wilcoxon rank-sum test, and selected significant drugs are shown in enlarged panels.

Supplementary Figure 11 Predicted drug sensitivity stratified by SAPCD2 expression. Predicted IC50 values were inferred from TCGA-ESCA transcriptomes. Patients were divided into high and low expression groups for SAPCD2. Each panel shows one drug, with violin/box plots comparing predicted IC50 between groups (lower IC50 indicates higher sensitivity); group differences were tested using a two-sided Wilcoxon rank-sum test, and selected significant drugs are shown in enlarged panels.View less
