# Supplementary material for: Histone chaperone-based stratification combined with two-sample Mendelian randomization identifies ADORA2B and SAPCD2 as prognostic biomarkers in esophageal cancer
Source: Front Oncol. 2026 Apr 13;16:1764927. doi: 10.3389/fonc.2026.1764927 (PMC13111002; doi:10.3389/fonc.2026.1764927)
Supplement: Supplementary Figure 1 — Prognostic HCRGs, HCRG-based clustering, clinicopathologic distributions, and KEGG enrichment. (A) Univariate Cox regression identifies eight prognosis-related hub HCRGs in TCGA-ESCA (p < 0.20). (B) Heatmap of hub HCRG expression with sample clustering and clinical annotations. (C) Distribution of clinicopathologic features across the two clusters (χ²/Fisher’s exact tests). (D) KEGG enrichment of the 1,742 common DEGs (see Supplementary Table 4 for full results). [file DataSheet1.zip › SupplementaryFigures_0208/Fig. S7.pdf]

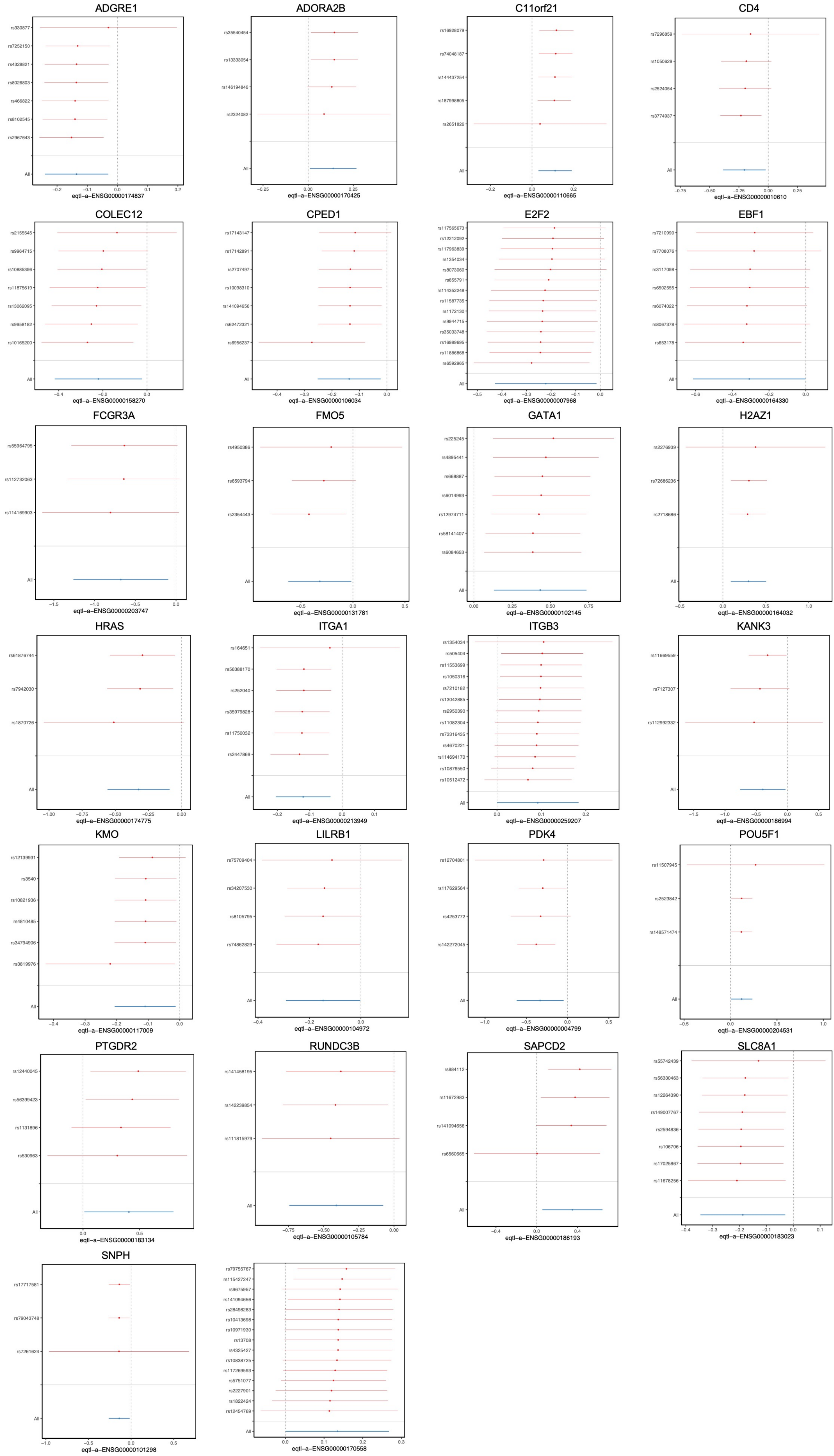

ADGRE1
rs330877
rs7252150
rs4328821
rs8026803
rs466822
rs8102545
rs2967643
All
eqtl-a-ENSG00000174837
ADORA2B
rs35540454
rs13333054
rs146194846
rs2324082
All
eqtl-a-ENSG00000170425
C11orf21
rs16928079
rs74048187
rs144437254
rs187998805
rs2651826
All
eqtl-a-ENSG00000110665
CD4
rs7296859
rs1050629
rs2524054
rs3774937
All
eqtl-a-ENSG00000010610
COLEC12
rs2155545
rs9964715
rs10885396
rs11875619
rs13062095
rs9958182
rs10165200
All
eqtl-a-ENSG00000158270
CPED1
rs17143147
rs17142891
rs2707497
rs10098310
rs141094656
rs62472321
rs6956237
All
eqtl-a-ENSG00000106034
E2F2
rs117565673
rs12212092
rs117963839
rs1354034
rs8073060
rs855791
rs114352248
rs11587735
rs1172130
rs9944715
rs35033748
rs16989695
rs11886868
rs6592965
All
eqtl-a-ENSG00000007968
EBF1
rs7210990
rs7708076
rs3117098
rs6502555
rs6074022
rs8067378
rs653178
All
eqtl-a-ENSG00000164330
FCGR3A
rs55964795
rs112732063
rs114169903
All
eqtl-a-ENSG00000203747
FMO5
rs4950386
rs6593794
rs2354443
All
eqtl-a-ENSG00000131781
GATA1
rs225245
rs4895441
rs668887
rs6014993
rs12974711
rs58141407
rs6084653
All
eqtl-a-ENSG00000102145
H2AZ1
rs2276939
rs72686236
rs2718686
All
eqtl-a-ENSG00000164032
HRAS
rs61876744
rs7942030
rs1870726
All
eqtl-a-ENSG00000174775
ITGA1
rs164651
rs56388170
rs252040
rs35979828
rs11750032
rs2447869
All
eqtl-a-ENSG00000213949
ITGB3
rs1354034
rs505404
rs11553699
rs1050316
rs7210182
rs13042885
rs2950390
rs11082304
rs73316435
rs4670221
rs114694170
rs10876550
rs10512472
All
eqtl-a-ENSG00000259207
KANK3
rs11669559
rs7127307
rs112992332
All
eqtl-a-ENSG00000186994
KMO
rs12139931
rs3540
rs10821936
rs4810485
rs34794906
rs3819976
All
eqtl-a-ENSG00000117009
LILRB1
rs75709404
rs34207530
rs8105795
rs74862829
All
eqtl-a-ENSG00000104972
PDK4
rs12704801
rs117629564
rs4253772
rs142272045
All
eqtl-a-ENSG00000004799
POU5F1
rs11507945
rs2523842
rs148571474
All
eqtl-a-ENSG00000204531
PTGDR2
rs12440045
rs56399423
rs1131896
rs530963
All
eqtl-a-ENSG00000183134
RUNDC3B
rs141458195
rs142239854
rs111815979
All
eqtl-a-ENSG00000105784
SAPCD2
rs884112
rs11672983
rs141094656
rs6560665
All
eqtl-a-ENSG00000186193
SLC8A1
rs55742439
rs56330463
rs12264390
rs149007767
rs2594836
rs106706
rs17025867
rs11678256
All
eqtl-a-ENSG00000183023
SNPH
rs17717581
rs79043748
rs7261624
All
eqtl-a-ENSG00000101298
rs79755767
rs115427247
rs9675957
rs141094656
rs28498283
rs10413698
rs10971930
rs13708
rs4325427
rs10838725
rs117269593
rs5751077
rs2227901
rs1822424
rs12454769
All
eqtl-a-ENSG00000170558
