# Supplementary material for: Histone chaperone-based stratification combined with two-sample Mendelian randomization identifies ADORA2B and SAPCD2 as prognostic biomarkers in esophageal cancer
Source: Front Oncol. 2026 Apr 13;16:1764927. doi: 10.3389/fonc.2026.1764927 (PMC13111002; doi:10.3389/fonc.2026.1764927)
Supplement: Supplementary Figure 1 — Prognostic HCRGs, HCRG-based clustering, clinicopathologic distributions, and KEGG enrichment. (A) Univariate Cox regression identifies eight prognosis-related hub HCRGs in TCGA-ESCA (p < 0.20). (B) Heatmap of hub HCRG expression with sample clustering and clinical annotations. (C) Distribution of clinicopathologic features across the two clusters (χ²/Fisher’s exact tests). (D) KEGG enrichment of the 1,742 common DEGs (see Supplementary Table 4 for full results). [file DataSheet1.zip › SupplementaryFigures_0208/Fig. S9.pdf]

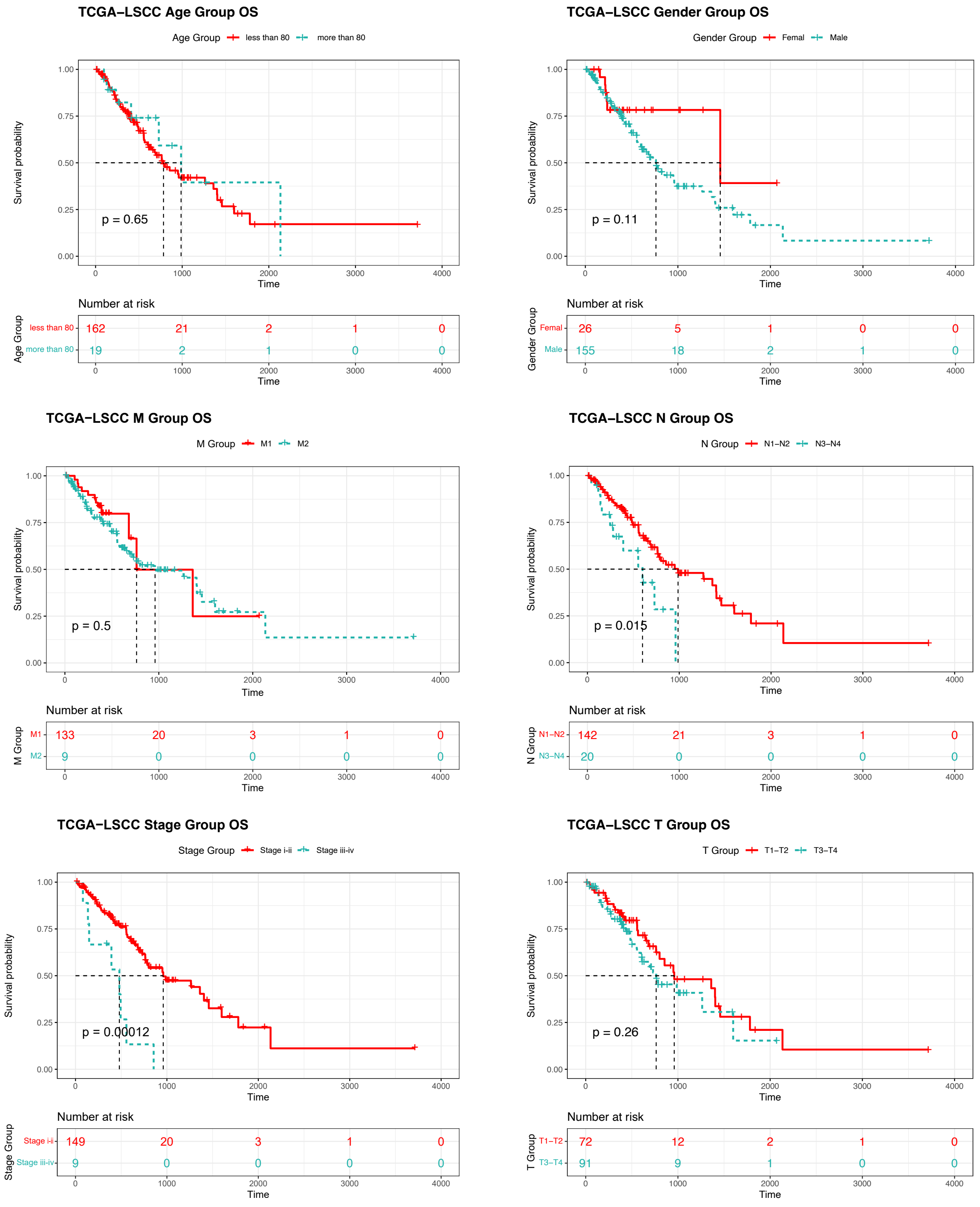
TCGA−LSCC Age Group OS
Age Group less than 80 more than 80
Survival probability
p = 0.65
Time
Number at risk
Age Group
less than 80 162 21 2 1 0
more than 80 19 2 1 0 0
TCGA−LSCC Gender Group OS
Gender Group Femal Male
p = 0.11
Gender Group
Femal 26 5 1 0 0
Male 155 18 2 1 0
TCGA−LSCC M Group OS
M Group M1 M2
p = 0.5
M Group
M1 133 20 3 1 0
M2 9 0 0 0 0
TCGA−LSCC N Group OS
N Group N1−N2 N3−N4
p = 0.015
N Group
N1−N2 142 21 3 1 0
N3−N4 20 0 0 0 0
TCGA−LSCC Stage Group OS
Stage Group Stage i-ii Stage iii-iv
p = 0.00012
Stage Group
Stage i-ii 149 20 3 1 0
Stage iii-iv 9 0 0 0 0
TCGA−LSCC T Group OS
T Group T1−T2 T3−T4
p = 0.26
T Group
T1−T2 72 12 2 1 0
T3−T4 91 9 1 0 0
