# Supplementary material for: Histone chaperone-based stratification combined with two-sample Mendelian randomization identifies ADORA2B and SAPCD2 as prognostic biomarkers in esophageal cancer
Source: Front Oncol. 2026 Apr 13;16:1764927. doi: 10.3389/fonc.2026.1764927 (PMC13111002; doi:10.3389/fonc.2026.1764927)
Supplement: Supplementary Figure 1 — Prognostic HCRGs, HCRG-based clustering, clinicopathologic distributions, and KEGG enrichment. (A) Univariate Cox regression identifies eight prognosis-related hub HCRGs in TCGA-ESCA (p < 0.20). (B) Heatmap of hub HCRG expression with sample clustering and clinical annotations. (C) Distribution of clinicopathologic features across the two clusters (χ²/Fisher’s exact tests). (D) KEGG enrichment of the 1,742 common DEGs (see Supplementary Table 4 for full results). [file DataSheet1.zip › SupplementaryFigures_0208/Fig. S8.pdf]

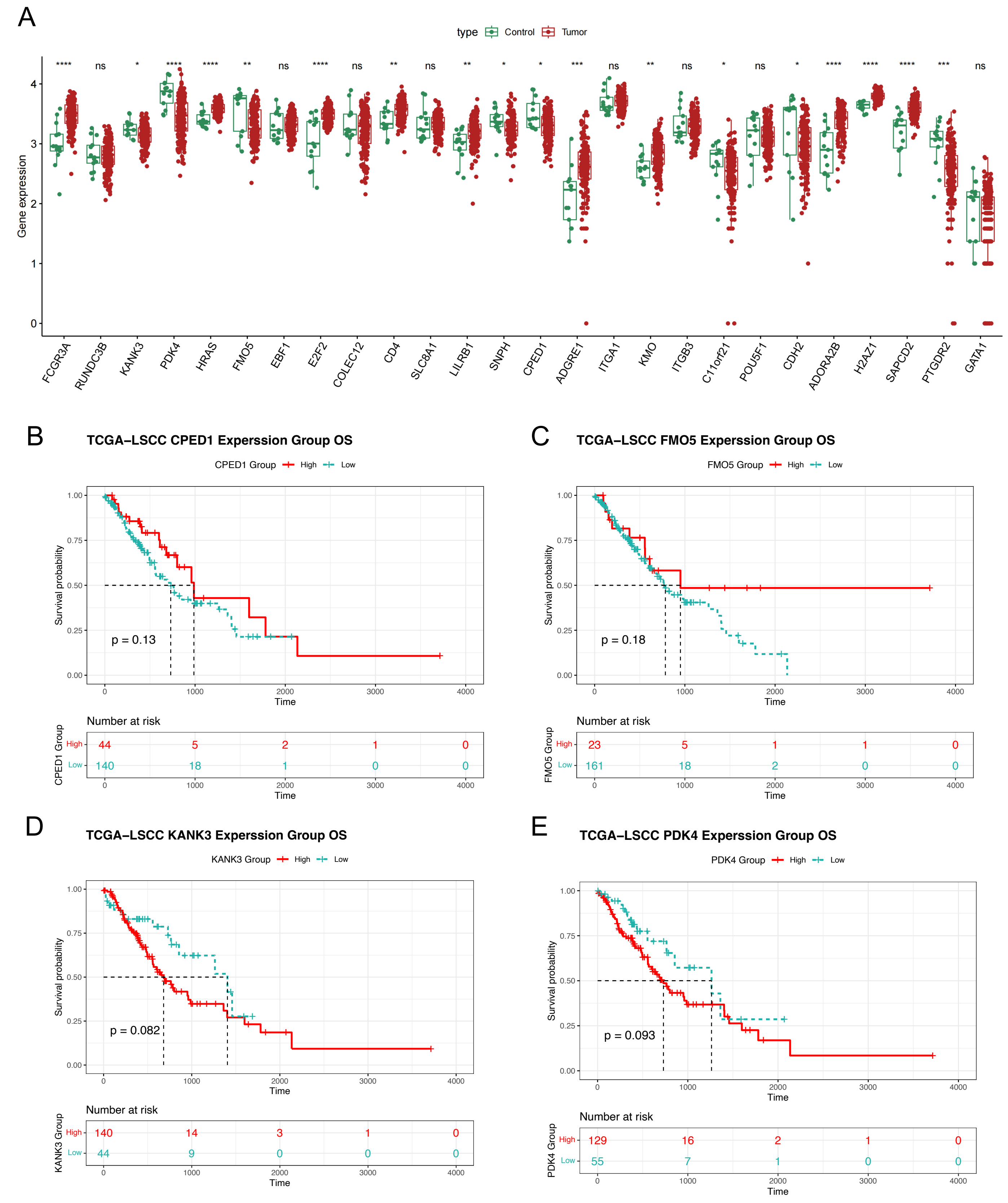
A
type Control Tumor
Gene expression
**** ns * **** **** ** ns **** ns ** ns ** * * *** ns ** ns * ns * **** **** **** *** ns
FCGR3A RUNDC3B KANK3 PDK4 HRAS FMO5 EBF1 E2F2 COLEC12 CD4 SLC8A1 LILRB1 SNPH CPED1 ADGRE1 ITGA1 KMO ITGB3 C11orf21 POU5F1 CDH2 ADORA2B H2AZ1 SAPCD2 PTGDR2 GATA1
B
TCGA–LSCC CPED1 Experssion Group OS
CPED1 Group High Low
Survival probability
p = 0.13
Time
Number at risk
CPED1 Group
High 44 5 2 1 0
Low 140 18 1 0 0
C
TCGA–LSCC FMO5 Experssion Group OS
FMO5 Group High Low
Survival probability
p = 0.18
Time
Number at risk
FMO5 Group
High 23 5 1 1 0
Low 161 18 2 0 0
D
TCGA–LSCC KANK3 Experssion Group OS
KANK3 Group High Low
Survival probability
p = 0.082
Time
Number at risk
KANK3 Group
High 140 14 3 1 0
Low 44 9 0 0 0
E
TCGA–LSCC PDK4 Experssion Group OS
PDK4 Group High Low
Survival probability
p = 0.093
Time
Number at risk
PDK4 Group
High 129 16 2 1 0
Low 55 7 1 0 0
