# Supplementary figures and images for: Histone chaperone-based stratification combined with two-sample Mendelian randomization identifies ADORA2B and SAPCD2 as prognostic biomarkers in esophageal cancer
Source: Front Oncol. 2026 Apr 13;16:1764927. doi: 10.3389/fonc.2026.1764927 (PMC13111002; doi:10.3389/fonc.2026.1764927)

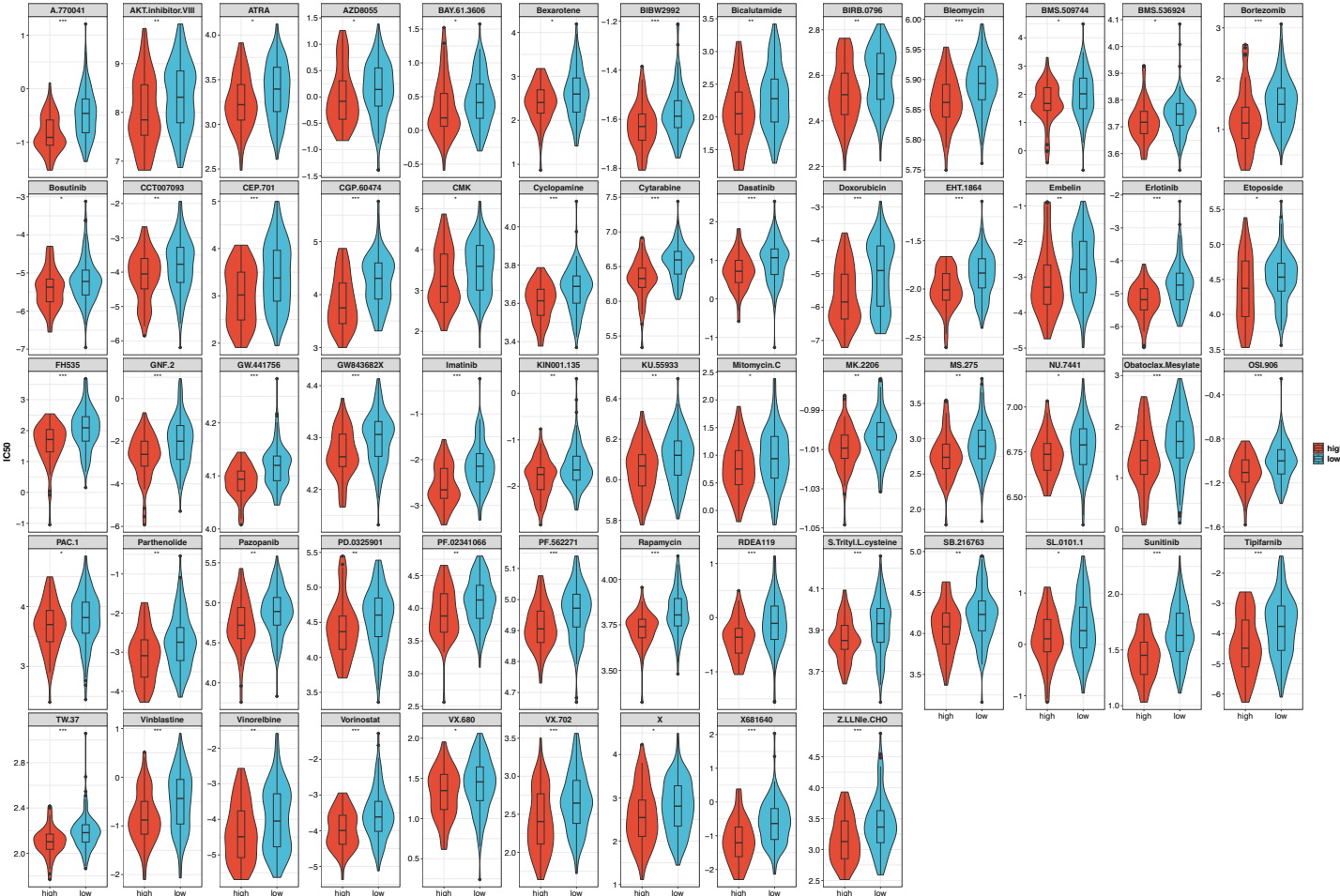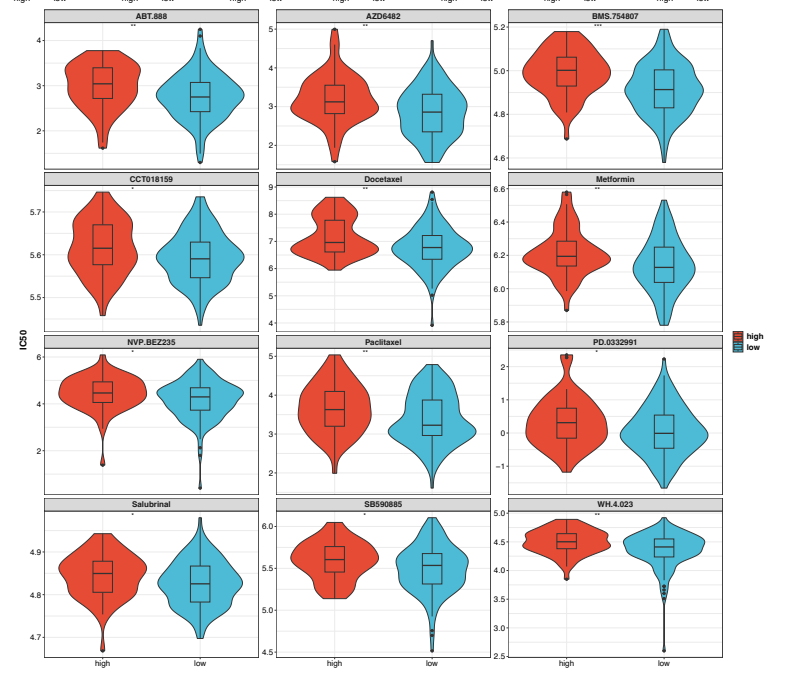

Supplement: Supplementary Figure 1 — Prognostic HCRGs, HCRG-based clustering, clinicopathologic distributions, and KEGG enrichment. (A) Univariate Cox regression identifies eight prognosis-related hub HCRGs in TCGA-ESCA (p < 0.20). (B) Heatmap of hub HCRG expression with sample clustering and clinical annotations. (C) Distribution of clinicopathologic features across the two clusters (χ²/Fisher’s exact tests). (D) KEGG enrichment of the 1,742 common DEGs (see Supplementary Table 4 for full results). [file DataSheet1.zip › SupplementaryFigures_0208/Fig. S11.pdf]

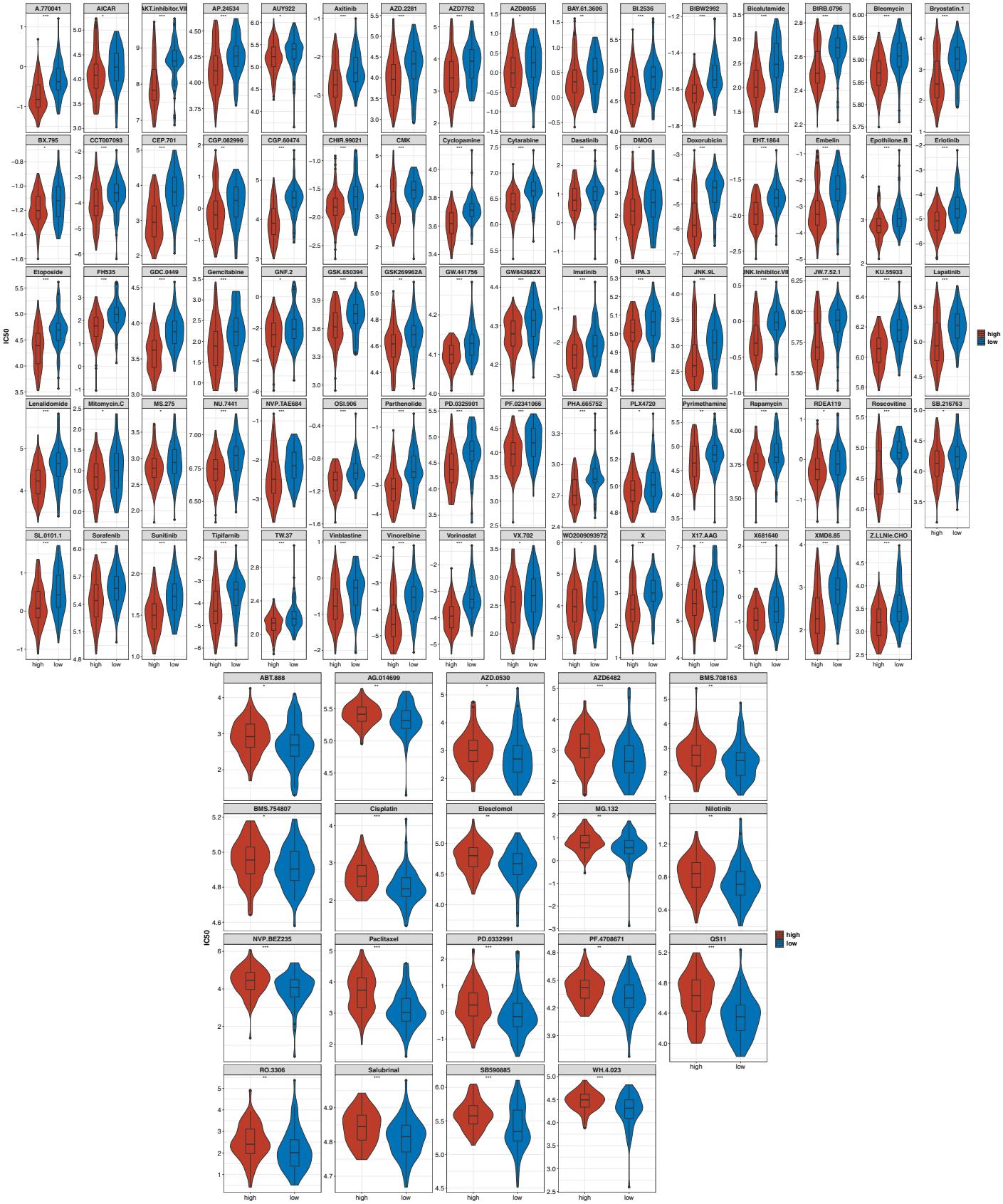

Supplement: Supplementary Figure 1 — Prognostic HCRGs, HCRG-based clustering, clinicopathologic distributions, and KEGG enrichment. (A) Univariate Cox regression identifies eight prognosis-related hub HCRGs in TCGA-ESCA (p < 0.20). (B) Heatmap of hub HCRG expression with sample clustering and clinical annotations. (C) Distribution of clinicopathologic features across the two clusters (χ²/Fisher’s exact tests). (D) KEGG enrichment of the 1,742 common DEGs (see Supplementary Table 4 for full results). [file DataSheet1.zip › SupplementaryFigures_0208/Fig. S10.pdf]

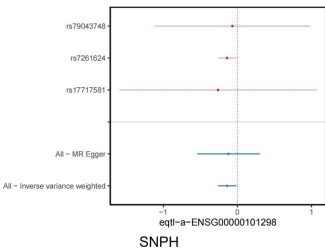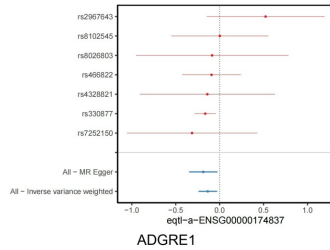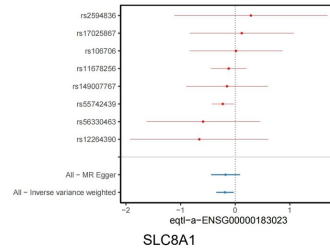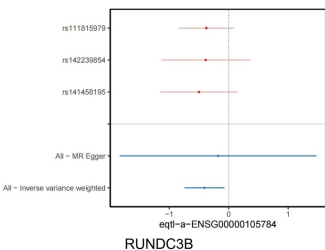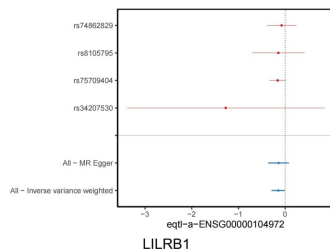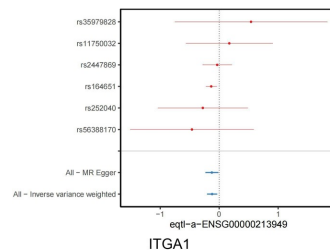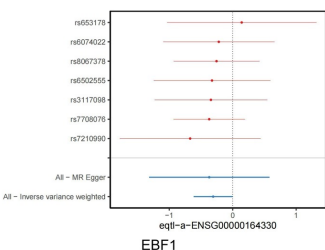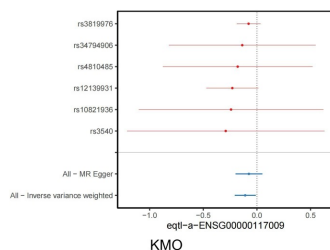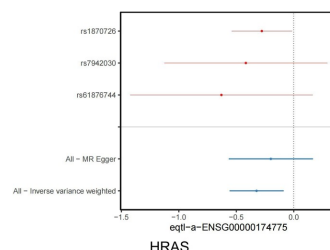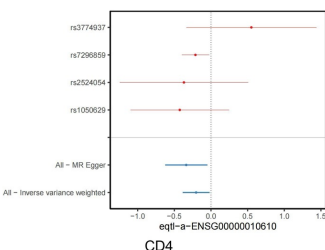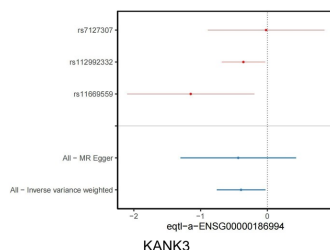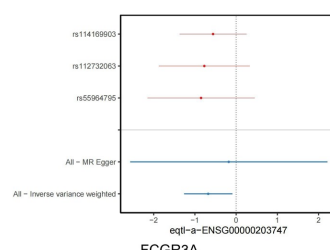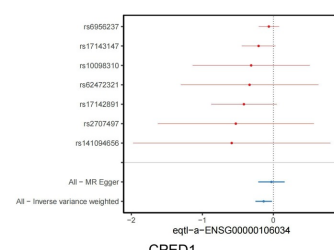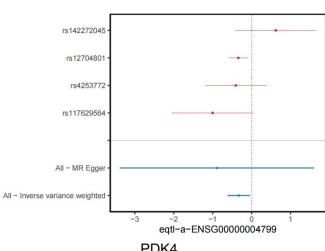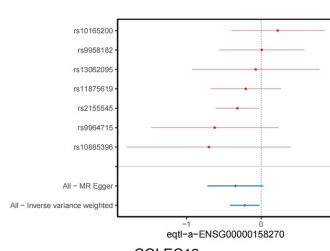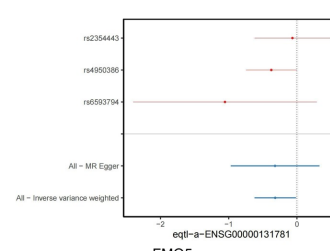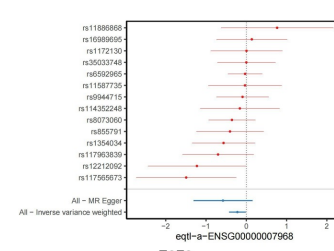

Supplement: Supplementary Figure 1 — Prognostic HCRGs, HCRG-based clustering, clinicopathologic distributions, and KEGG enrichment. (A) Univariate Cox regression identifies eight prognosis-related hub HCRGs in TCGA-ESCA (p < 0.20). (B) Heatmap of hub HCRG expression with sample clustering and clinical annotations. (C) Distribution of clinicopathologic features across the two clusters (χ²/Fisher’s exact tests). (D) KEGG enrichment of the 1,742 common DEGs (see Supplementary Table 4 for full results). [file DataSheet1.zip › SupplementaryFigures_0208/Fig. S5.pdf]

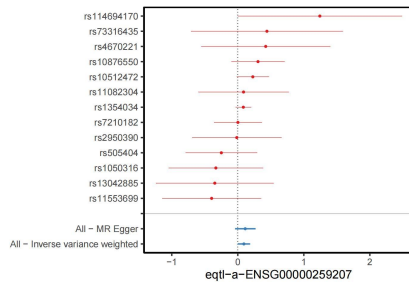

ITGB3

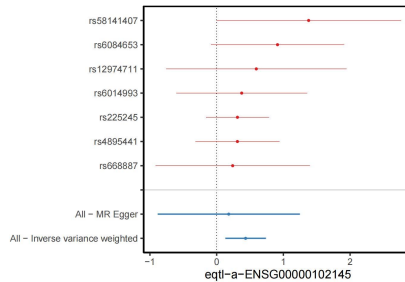

GATA1

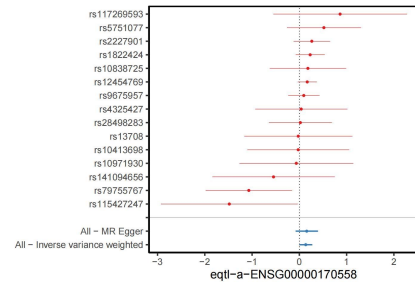

CDH2

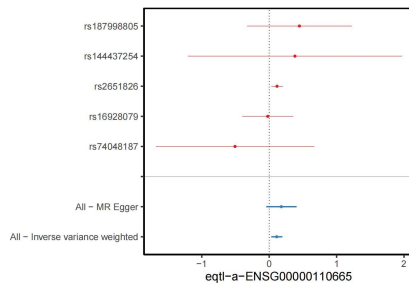

C11orf21

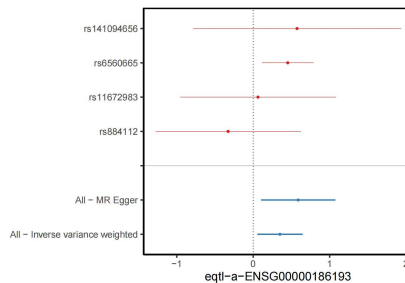

SAPCD2

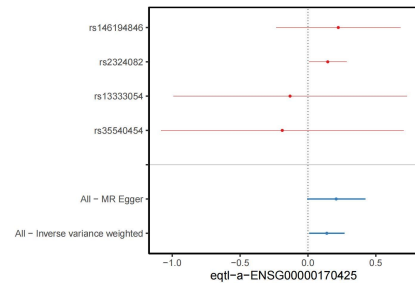

ADORA2B

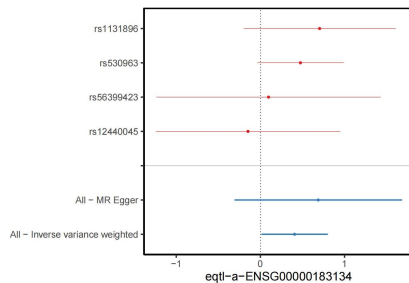

PTGDR2

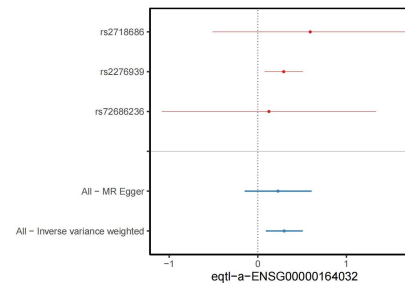

H2AZ1

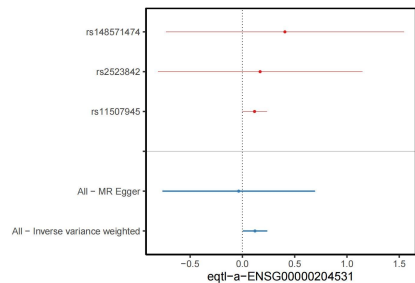

POU5F1

Supplement: Supplementary Figure 1 — Prognostic HCRGs, HCRG-based clustering, clinicopathologic distributions, and KEGG enrichment. (A) Univariate Cox regression identifies eight prognosis-related hub HCRGs in TCGA-ESCA (p < 0.20). (B) Heatmap of hub HCRG expression with sample clustering and clinical annotations. (C) Distribution of clinicopathologic features across the two clusters (χ²/Fisher’s exact tests). (D) KEGG enrichment of the 1,742 common DEGs (see Supplementary Table 4 for full results). [file DataSheet1.zip › SupplementaryFigures_0208/Fig. S4.pdf]

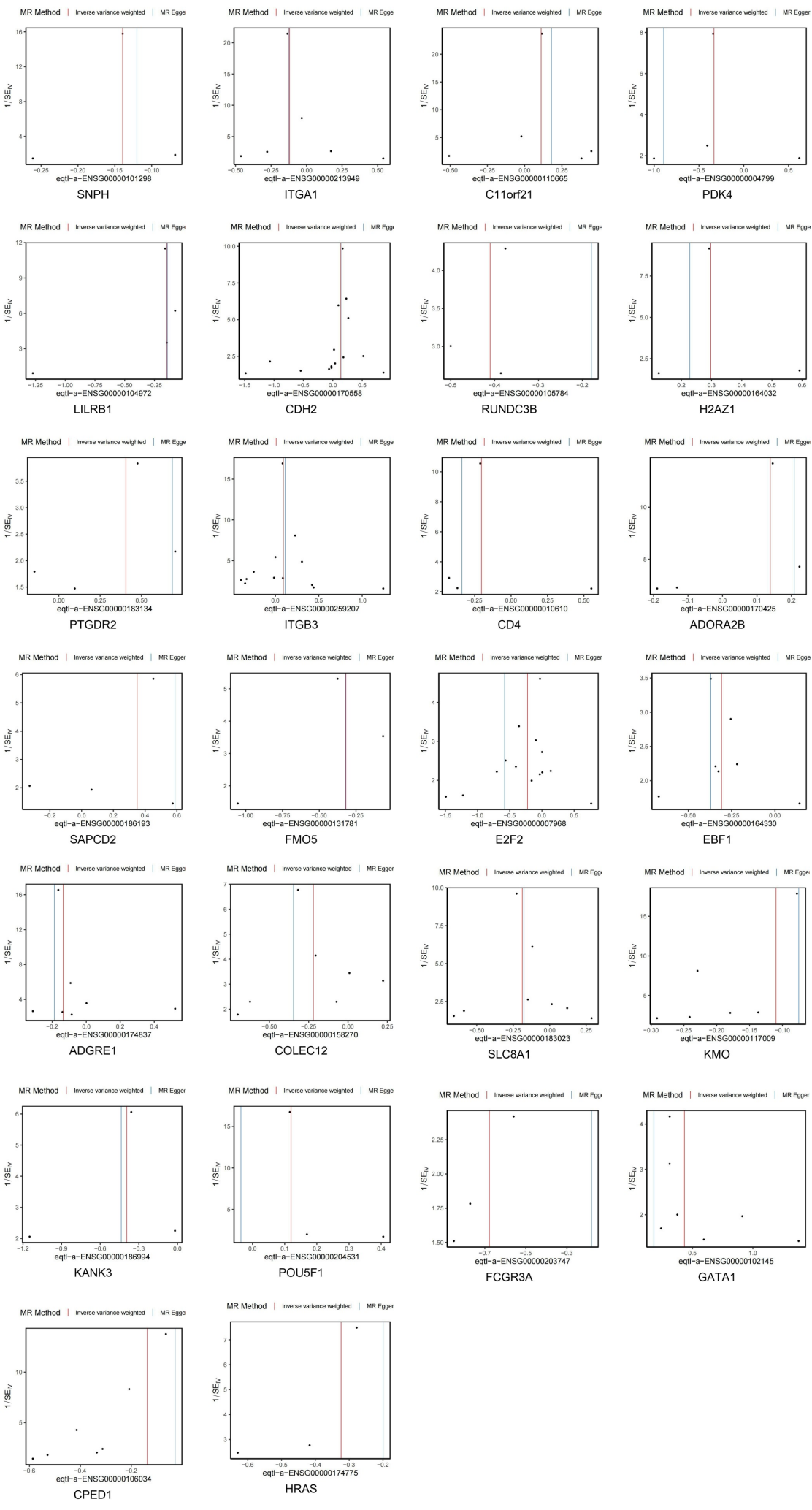

Supplement: Supplementary Figure 1 — Prognostic HCRGs, HCRG-based clustering, clinicopathologic distributions, and KEGG enrichment. (A) Univariate Cox regression identifies eight prognosis-related hub HCRGs in TCGA-ESCA (p < 0.20). (B) Heatmap of hub HCRG expression with sample clustering and clinical annotations. (C) Distribution of clinicopathologic features across the two clusters (χ²/Fisher’s exact tests). (D) KEGG enrichment of the 1,742 common DEGs (see Supplementary Table 4 for full results). [file DataSheet1.zip › SupplementaryFigures_0208/Fig. S6.pdf]

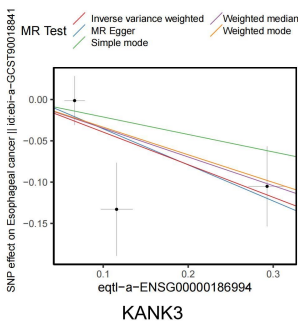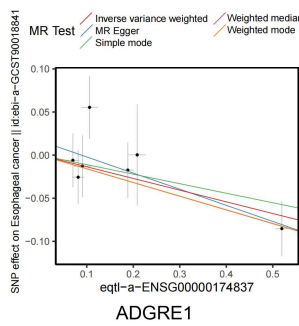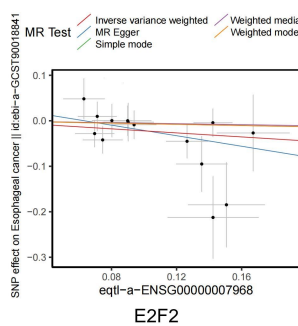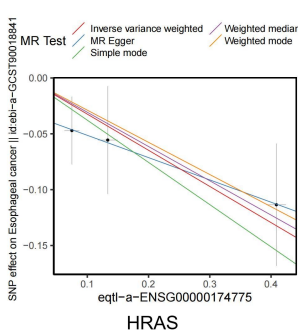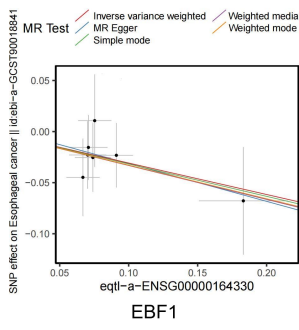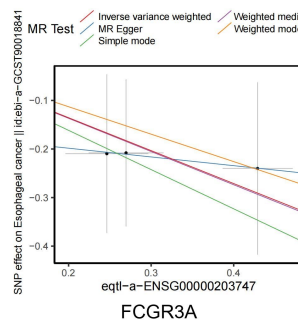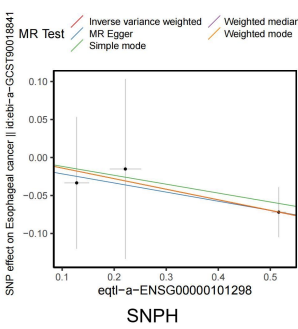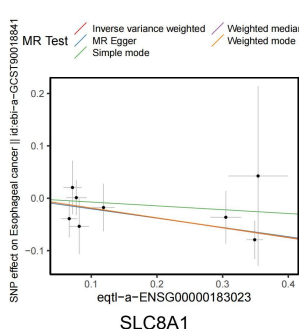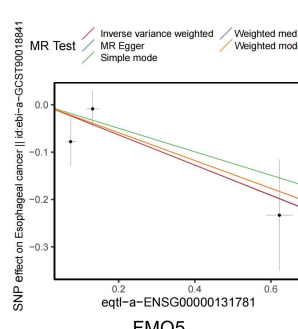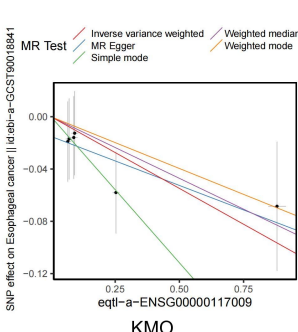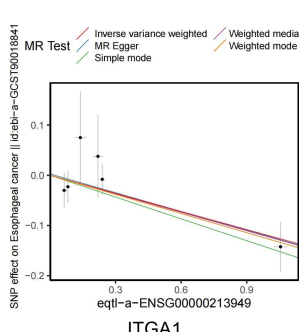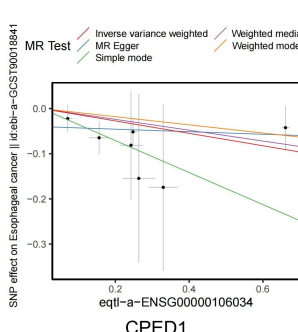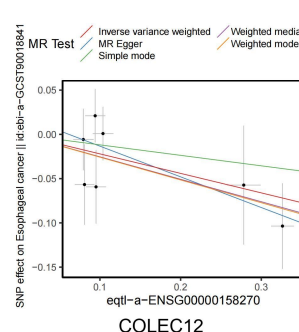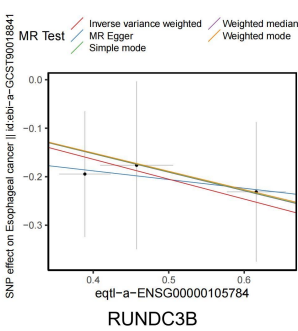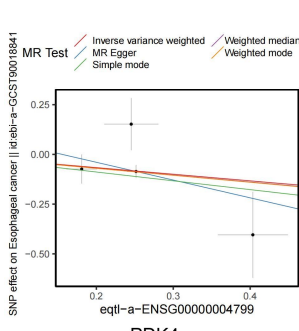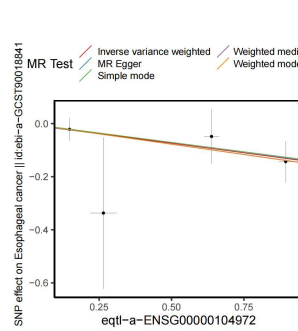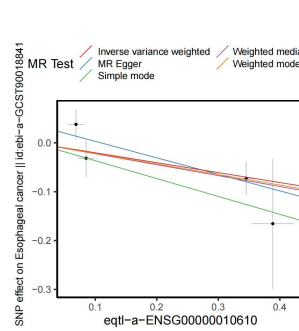

Supplement: Supplementary Figure 1 — Prognostic HCRGs, HCRG-based clustering, clinicopathologic distributions, and KEGG enrichment. (A) Univariate Cox regression identifies eight prognosis-related hub HCRGs in TCGA-ESCA (p < 0.20). (B) Heatmap of hub HCRG expression with sample clustering and clinical annotations. (C) Distribution of clinicopathologic features across the two clusters (χ²/Fisher’s exact tests). (D) KEGG enrichment of the 1,742 common DEGs (see Supplementary Table 4 for full results). [file DataSheet1.zip › SupplementaryFigures_0208/Fig. S3.pdf]

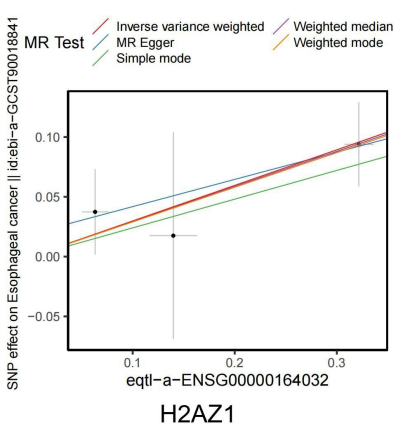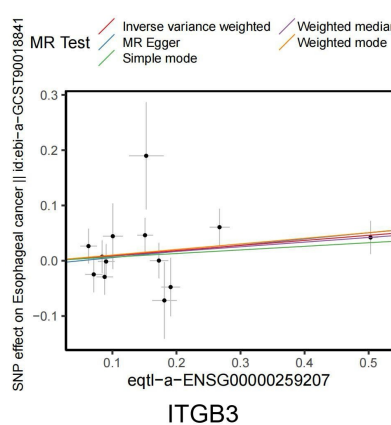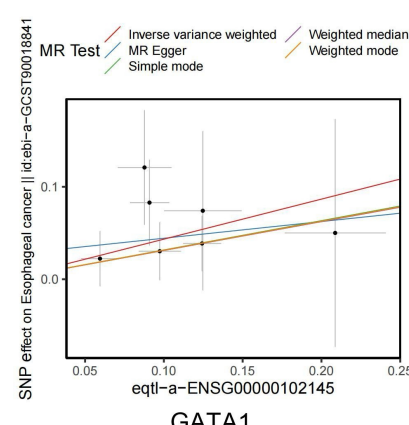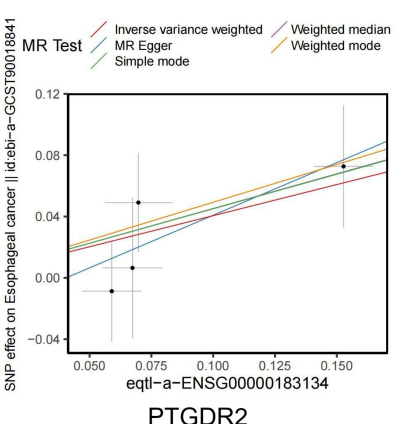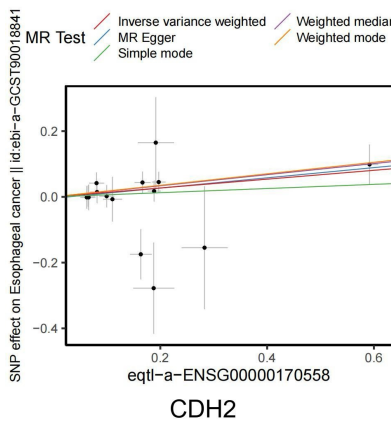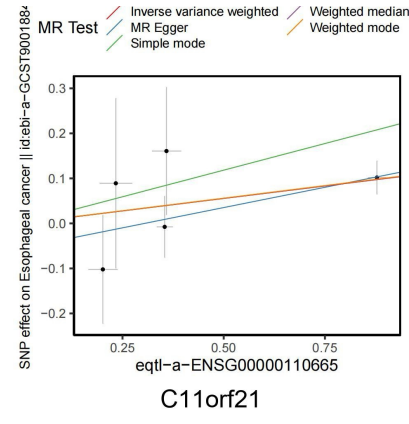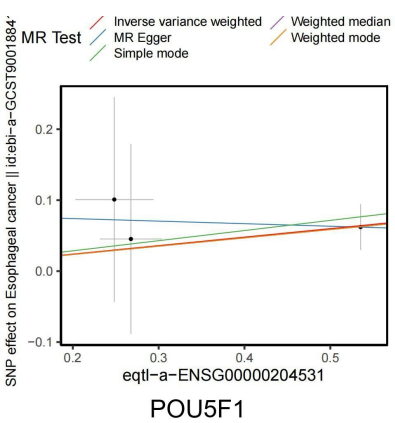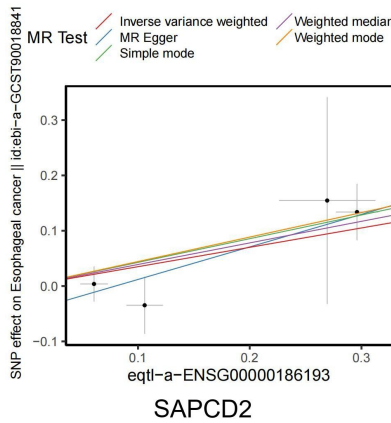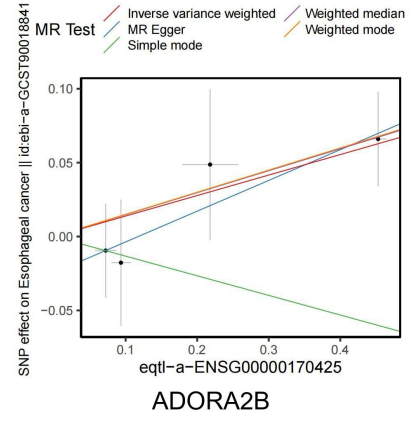

Supplement: Supplementary Figure 1 — Prognostic HCRGs, HCRG-based clustering, clinicopathologic distributions, and KEGG enrichment. (A) Univariate Cox regression identifies eight prognosis-related hub HCRGs in TCGA-ESCA (p < 0.20). (B) Heatmap of hub HCRG expression with sample clustering and clinical annotations. (C) Distribution of clinicopathologic features across the two clusters (χ²/Fisher’s exact tests). (D) KEGG enrichment of the 1,742 common DEGs (see Supplementary Table 4 for full results). [file DataSheet1.zip › SupplementaryFigures_0208/Fig. S2.pdf]

A

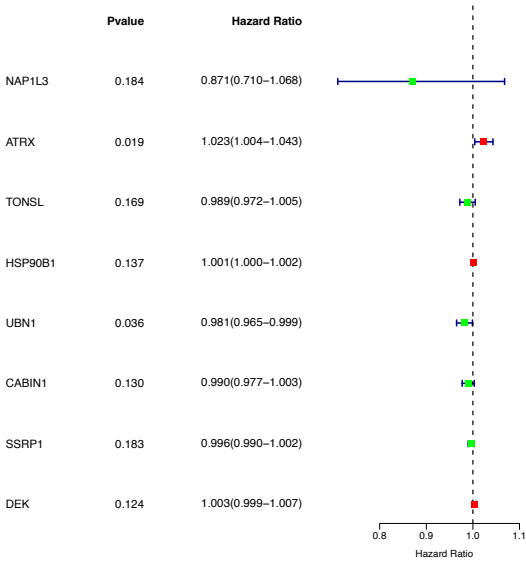

B

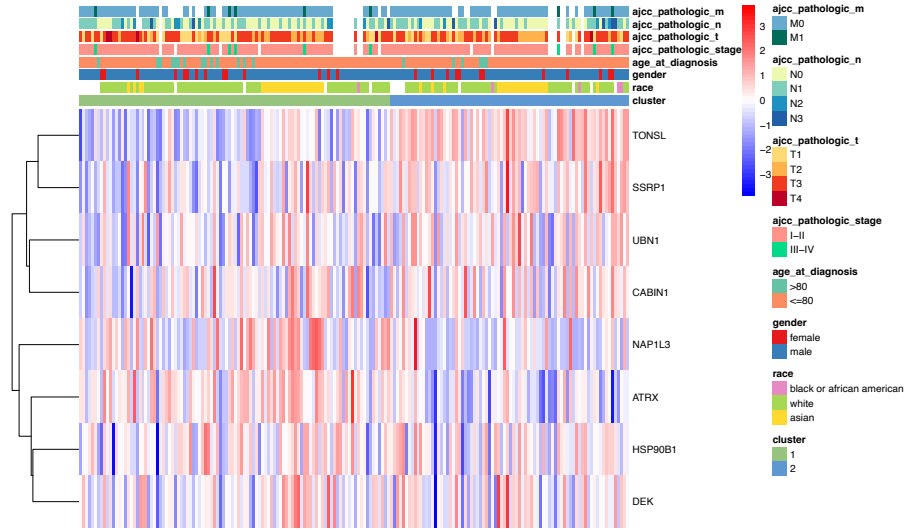

C

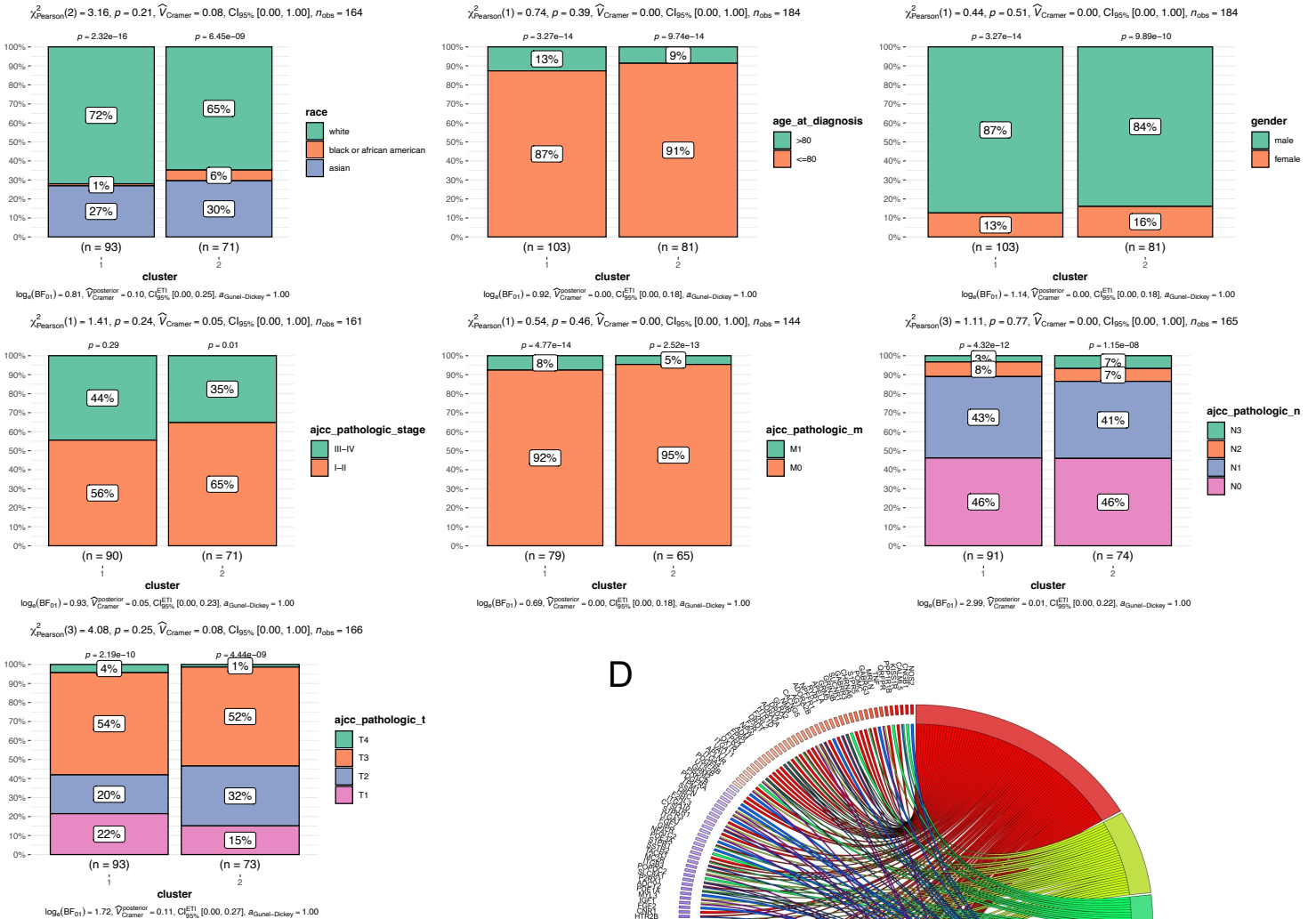

D

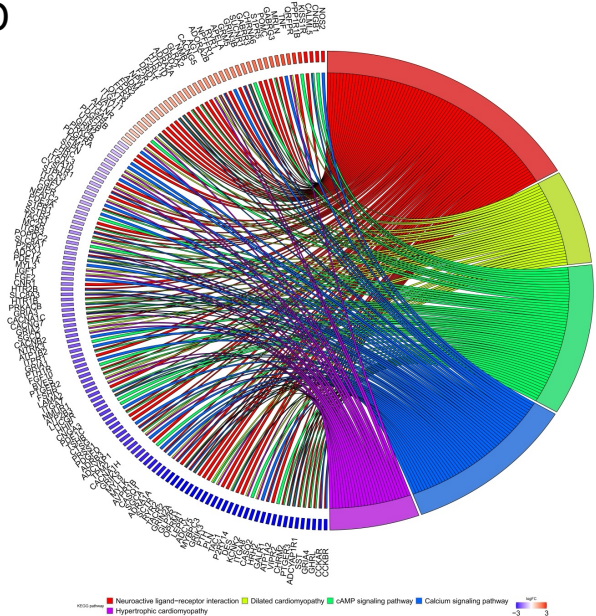

Supplement: Supplementary Figure 1 — Prognostic HCRGs, HCRG-based clustering, clinicopathologic distributions, and KEGG enrichment. (A) Univariate Cox regression identifies eight prognosis-related hub HCRGs in TCGA-ESCA (p < 0.20). (B) Heatmap of hub HCRG expression with sample clustering and clinical annotations. (C) Distribution of clinicopathologic features across the two clusters (χ²/Fisher’s exact tests). (D) KEGG enrichment of the 1,742 common DEGs (see Supplementary Table 4 for full results). [file DataSheet1.zip › SupplementaryFigures_0208/Fig. S1.pdf]
